# Multi-dimensional data integration algorithm based on random walk with restart

**DOI:** 10.1186/s12859-021-04029-3

**Published:** 2021-02-27

**Authors:** Yuqi Wen, Xinyu Song, Bowei Yan, Xiaoxi Yang, Lianlian Wu, Dongjin Leng, Song He, Xiaochen Bo

**Affiliations:** 1 Department of Biotechnology, Beijing Institute of Radiation Medicine, Beijing, 100850 People’s Republic of China; 2 Department of Biomedical Engineering, Chinese PLA General Hospital, Beijing, 100853 People’s Republic of China; 3 Experimental Center, Beijing Friendship Hospital, Capital Medical University, Beijing, 100069 People’s Republic of China; 4 Academy of Medical Engineering and Translational Medicine, Tianjin University, Tianjin, 300072 People’s Republic of China

**Keywords:** Random walk with restart, Multiplex network, Multi-dimensional data integration, Cancer subtyping

## Abstract

**Background:**

The accumulation of various multi-omics data and computational approaches for data integration can accelerate the development of precision medicine. However, the algorithm development for multi-omics data integration remains a pressing challenge.

**Results:**

Here, we propose a multi-omics data integration algorithm based on random walk with restart (RWR) on multiplex network. We call the resulting methodology Random Walk with Restart for multi-dimensional data Fusion (RWRF). RWRF uses similarity network of samples as the basis for integration. It constructs the similarity network for each data type and then connects corresponding samples of multiple similarity networks to create a multiplex sample network. By applying RWR on the multiplex network, RWRF uses stationary probability distribution to fuse similarity networks. We applied RWRF to The Cancer Genome Atlas (TCGA) data to identify subtypes in different cancer data sets. Three types of data (mRNA expression, DNA methylation, and microRNA expression data) are integrated and network clustering is conducted. Experiment results show that RWRF performs better than single data type analysis and previous integrative methods.

**Conclusions:**

RWRF provides powerful support to users to decipher the cancer molecular subtypes, thus may benefit precision treatment of specific patients in clinical practice.

## Background

The rapid development of biotechnology enables researchers to generate multiple types of biomedical data. For example, The Cancer Genome Atlas (TCGA) has accumulated comprehensive multi-omics molecular profiles of 33 cancer types from more than 10,000 patients (e.g., genomics, transcriptomics, proteomics, and epigenomics) [1]. The integrated analysis of multi-omics data not only help to explore disease-related biological processes through differences in omics features between different patients [2, 3], but also contributes to precision treatment of specific patients in clinical practice.

Compared with methods that use only a single data type, data integration approach enables more comprehensive and informative analysis of biomedical data. Integrating multiple data types can compensate for missing or unreliable information in any single data type, and multiple sources of evidence pointing to the same result are less likely to lead to false positives. Therefore, algorithms for integrating multi-omics or multi-dimensional biomedical data become indispensable key technologies for multi-omics research and new algorithms are increasingly needed.

One of the research areas most in need of data integration approach is cancer subtyping. For cancer subtyping, there are many computational strategies based on data integration. The simplest way is to concatenate multiple features from different omics data. But concatenation approach dilutes the already low signal-to-noise ratio in specific data type [4], thus affecting the accuracy of subtyping. The second strategy is to cluster each type of omics data separately and then integrate these different clusters based on the similarity of clustering results. Previously, after clustering of each type of omics data, data integration mainly relies on experts’ artificial integration, which are inefficient and expensive. Afterward there comes a method called consensus clustering or cluster ensemble [5]. The advantage of this method is that it can integrate clustering results through algorithm and obtain common information across various types of data. But how to measure the consistency between clustering results and how to use valuable complementary information are problems that need to be solved. A typical consensus clustering method is cluster-of-cluster assignments (COCA). The Cancer Genome Atlas Network et al. used COCA to integrate five types of omics data for subtyping of breast cancer [6]. The algorithm takes as input the binary vectors that represent each of the platform-specific cluster groups and reclusters the samples according to those vectors [7]. It is useful but is less powerful when the molecular patterns are not strong enough to specify a distinct group on multiple individual platforms [8]. The third strategy is to use co-projection approaches. To find common low-dimensional subspace across different data types, co-projection (joint dimensionality reduction) methods are efficient approaches [9–18]. Co-projection methods came into focus of data integration analysis due to their prominent ability to integrate large-scale, diverse, and heterogeneous biomedical data. For example, Shen et al. proposed iCluster, a joint latent variable model for data integration [14, 19, 20]. It uses a probabilistic matrix factorization approach to simultaneously decompose data matrices, representing different data types over the same number of samples, into a common feature space [21]. Katherine et al. performed integrative molecular subtyping with iCluster using four data types (copy number, DNA methylation, mRNA, and miRNA) across 9759 tumor samples, identifying 28 Clusters [8]. Although it is powerful, iCluster and other similar co-projection methods that operate with high-dimensional feature × sample matrices have scalability drawbacks, making these methods sensitive to gene preselection step [4, 10, 22]. Instead of processing largescale matrices constructed over a large number of features, network-based methods use samples network as a basis for integration. Wang et al. proposed a network-based method called similarity network fusion (SNF) [4]. SNF is shown to be effective in cancer subtyping. But SNF only uses local topology information of sample similarity network, thus its integration performance is limited. Affinity Network Fusion (ANF), an “upgrade” of SNF which incorporates weights of each view, also has the same problem [23].

In this paper, we proposed a multi-omics data integration algorithm based on random walk with restart (RWR) on multiplex network. RWR algorithm can be seen as an extension of the PageRank algorithm developed by Google [24]. An imaginary particle starts a random walk from the seed node. At each step, the particle either walks to other nodes with a certain probability, or returns to the seed node. After several iterations, the stationary probability distribution can be seen as the distribution of influence of the seed node. RWR algorithm has been applied to the recommendation system and has obtained good performance [25]. For inference of novel biological relations such as drug-target interaction and disease-gene association prediction, RWR based on the heterogeneous network has also gotten excellent prediction performance [26–29].

We extend RWR to integrate similarity networks established from multiple omics data. Two methods called Random Walk with Restart for multi-dimensional data Fusion (RWRF) and Random Walk with Restart and Neighbor information-based multi-dimensional data Fusion (RWRNF) are proposed. The methods consist of two main steps: (1) Construction of sample similarity network for each data type and construction of a multiplex network in which corresponding samples of multiple similarity networks are connected. (2) Random walk with restart (RWR) on the multiplex network. After several iterations, the stationary probability distribution can be obtained. The stationary probability distribution is utilized to get the integrated similarity network, which contains each type of data’s information.

Our method can not only capture the consistent patterns across different omics data, but also make use of complementary information provided by different omics data. They can automatically capture various structure information and make full use of topology information of the whole similarity network of each type of data. Our methods have strong anti-noise ability without being affected by an increase in the number of features. Moreover, by introducing the random walk theory, our methods have a clear interpretation.

We applied our methods to TCGA data to identify subtypes in different cancer data sets. Three types of omics data are integrated and network clustering (subtyping) is conducted. Experiment results show that our methods perform better than previous methods. The subtyping results also provide an analytical basis for clinical applications. Source code is available at https://github.com/Sepstar/RWRF/.

## Results

### Overview

RWRF and RWRNF are two multi-omics data integration algorithms based on random walk with restart on multiplex network. Given two or more types of omics data, the two algorithms first construct similarity network for each omics data based on the same batch of samples. Then corresponding samples of multiple similarity networks are connected to construct a multiplex network. Inspired by the PageRank algorithm, we apply random walk with restart (RWR) to the multiplex network.

**Fig. 1:**
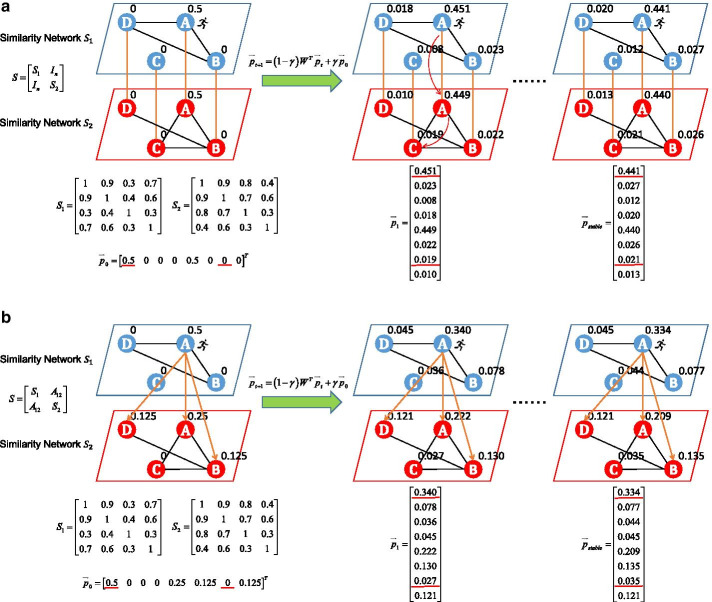
RWRF and RWRNF overview. **a** We constructed a multiplex network in which corresponding samples of $S_{1}$ and $S_{2}$ are connected. An imaginary particle starts a random walk from seed node A. The number next to the node indicates the probability of walking to the node. After several iterations, the stationary probability distribution will be obtained. RWRF utilizes the stationary probability distribution representing similarities between A and other nodes in the multiplex network. Here, we set *α* = 0.5. **b** Unlike RWRF, RWRNF connects $S_{1}$ and $S_{2}$ differently. For the sake of clarity and brevity, we only draw the connections from A in $S_{1}$ to other nodes in $S_{2}$. Here, we set *m* = 2, *α* = 0.5, *β* = 0.5. Note that edges with similarity less than 0.5 are omitted

For example, we have a similarity network ${S}_{1}$ constructed based on omics data 1 and a similarity network ${S}_{2}$ constructed based on omics data 2. As shown in Fig. 1a, corresponding samples are connected. Suppose that an imaginary particle starts a random walk from seed node A in the multiplex network. After several iterations, the stationary probability distribution ${\overrightarrow{p}}_{stable}$ represents similarities between A and other nodes in the multiplex network. Because of the different topology structure between ${S}_{1}$ and ${S}_{2}$, when the particle moves through the multiplex network, not only topology information of the similarity network constructed based on omics data 1 but also that of the similarity network constructed based on omics data 2 is used. The stationary probability distribution ${\overrightarrow{p}}_{stable}$ reflects the influence distribution of the seed node A based on the two omics data. For example, in similarity network ${S}_{1}$, the connection between A and C cannot be captured directly, but in the multiplex network, it can be obtained in just two steps by the random walk process. At last, based on the stationary distribution, two new similarity networks are fused to get the integrated similarity network, which contains each type of data’s information. Figure 1b illustrates the RWRNF process in the same way.

Making full use of topology information of each similarity network, the two algorithms have strong anti-noise ability. Aiming at tumor subtyping, the algorithms are applied to multi-omics cancer data from TCGA. By comparing with other algorithms, our algorithms are shown to be effective in cancer subtyping.

### Experimental setup

#### Datasets and preprocessing

In this paper, we applied our methods to 6 different cancer data sets from TCGA: adrenocortical carcinoma (ACC), bladder urothelial carcinoma (BLCA), head and neck squamous cell carcinoma (HNSC), uveal melanoma (UVM), pancreatic adenocarcinoma (PAAD) and thyroid carcinoma (THCA). For each of cancer data sets, we used 3 types of omics data: mRNA expression, DNA methylation, and microRNA (miRNA) expression.

First of all, we preprocess the data in the following steps:

1. Samples with all three types of omics data are retained.
2. If a sample has more than 20% data missed or more than 20% of samples do not measure a feature, we will delete the sample or the feature.
3. For each sample’s features, we fill the missing values with the mean value of the same feature in the other samples.
4. We normalized each row (each feature) of feature-sample matrix data with average 0 and standard deviation 1.

After four steps of data preprocessing, we obtained 76 ACC samples, 396 BLCA samples, 469 HNSC samples, 80 UVM samples, 175 PAAD samples, and 492 THCA samples.

#### Similarity measurement

We used the following formula to calculate the similarity measure between samples $C_{x}$ and $C_{y}$:

1 $$S(c_{x} ,c_{y} ) = \exp \left( { - \frac{{dist^{2} (c_{x} ,c_{y} )}}{{\mu E(c_{x} ,c_{y} )}}} \right)$$

where $dist(c_{x} ,c_{y} )$ is the Euclidean distance between sample $c_{x}$ and $c_{y}$, $\mu$ is a hyper-parameter. $E(c_{x} ,c_{y} )$ is defined as:

2 $$E(c_{x} ,c_{y} ) = \frac{{mean\left( {dist\left( {c_{x} ,N_{x} } \right)} \right) + mean\left( {dist\left( {c_{y} ,N_{y} } \right)} \right) + dist\left( {c_{x} ,c_{y} } \right)}}{3}$$

where $mean(dist(c_{x} ,N_{x} ))$ is the average value of the distances between $c_{x}$ and each of its $N$ neighbors. ${N}_{x}$ represents the neighbor nodes of ${c}_{x}$. According to Wang et al*.* study [4], we set $N{ = }20$, $\mu { = }0.5$.

#### Previous methods for comparison

We choose six previous methods designed for multi-dimensional data integration: concatenation, COCA, iCluster, intNMF, SNF and ANF. The concatenation method is a commonly used method that is simple and has a low computational cost. For each sample, the concatenation method assembles the multiple data type to a long vector, reserves complete information about multi-dimensional data, and treats the assembling vector as a new data type [30]. COCA takes as input the binary vectors that represent each of the platform-specific cluster groups and reclusters the samples according to those vectors [7]. iCluster is a joint latent variable model for data integration. This method models the tumor subtypes as unobserved latent variables which are simultaneously estimated from the multiple data types [31]. For iCluster, we used the 5% features with the largest median absolute deviation for each type of omics data. This is for the purpose of selecting the most informative genes for class detection [32]. Like iCluster, intNMF is a joint dimensionality reduction method and is an extension of non-Negative Matrix Factorization (NMF) [22]. Cantini et al. benchmarked joint multi-omics dimensionality reduction approaches for cancer subtyping [10]. They observed that intNMF performs best in clustering. For both iCluster and intNMF, we provide feature matrices as inputs. SNF method constructs sample similarity network for each data type and integrates these networks into a fused network using a nonlinear combination method [4]. Different from the linear integration strategy based on simple average or maximization, the network fusion step of SNF iteratively updates each of the networks with information from the other networks, making them more similar with each step. ANF, which incorporates weights of each view, is an “upgrade” of SNF [23].

#### Network clustering

We used spectral clustering on the sample similarity network for subtyping. The cluster number is the majority of the optimal number determined by seven cluster validity indexes: Ratkowsky Lance, Tau, Silhouette, C-index, SD-scat, SD-Dis, and Calinski-Harabasz (Additional file 1). Each indicator gives an optimal number of clusters. The two most voted cluster numbers are recommended.

#### Evaluation metrics

Two metrics are adopted for algorithm evaluation. First, we use the *Dunn* index to evaluate the quality of clustering results (subtyping results). The *Dunn* index is defined as follows:

3 $$DI=\underset{{C}_{k}\in C}{min}\left(\underset{{C}_{l}\in C}{min}\frac{dist({C}_{k},{C}_{l})}{\underset{{C}_{m}\in C}{max}diam({C}_{m})}\right)$$

where *C* is the collection of all clusters, $diam({C}_{m})$ is the largest intra-cluster distance in Cluster ${C}_{m}$, $dist({C}_{k},{C}_{l})$ is the distance between the nearest pair of samples in Cluster ${C}_{k}$ and Cluster ${C}_{l}$. The *Dunn* index has a value between zero and infinity, and should be maximized.

Besides, *P* value for the log-rank test of survival analysis is used to evaluate the significance of the difference between cancer subtypes. Statistical significance threshold is set to 0.05. If the *P* value is less than 0.05, it indicates a significant difference between the population survival curves representing different subtypes of cancer. This metric should be minimized.

### Performance evaluation

To prove the effectiveness of RWRF and RWRNF, we evaluated their performance by identifying subtypes in each of six different cancer data sets.

#### Comparison with single omics data-based model

**Fig. 2:**
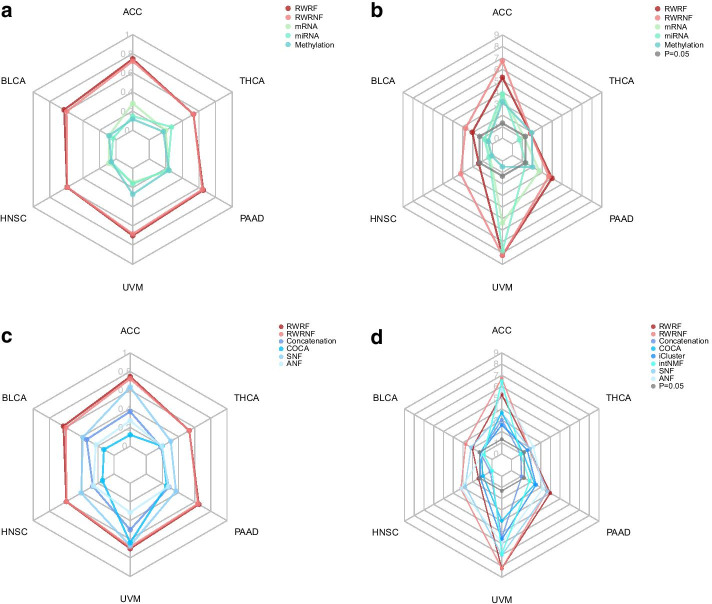
Performance comparison. **a** *Dunn* index comparison before and after the fusion in six different cancer data set. **b** *P* value for log-rank test comparison before and after the fusion in six different cancer data set. **c** *Dunn* index comparison with Concatenation, COCA, SNF and ANF in six different cancer data set. **d** *P* value for log-rank test comparison with Concatenation, COCA, iCluster, intNMF and two similarity-based methods: SNF and ANF in six different cancer data set. Here, *P* value for log-rank test is after − lg10

First, we compared data fusion by using our methods to the use of individual omics data types separately across the six different cancer data. For individual omics data type, sample similarity network is constructed and then clustered by spectral clustering. For RWRF and RWRNF, the added network fusion step is conducted before spectral clustering. Two measures (*Dunn* index and *P* value for log-rank test) are calculated to compare the subtyping results of individual omics data analysis and multi-omics data fusion. As shown in Fig. 2a, b (also shown in Additional file 2: Table S1, Additional file 3: Table S2), RWRF and RWRNF perform much better than individual omics data analysis. Data fusion by using our methods shows good subtyping results in all six cancer data sets.

#### Comparison with previous methods

Furthermore, to better evaluate the network fusion of RWRF and RWRNF, we compared our methods with six previous methods. Performance comparison was implemented across six different cancer data. The *Dunn* index comparison is shown in Fig. 2c, the *P* value comparison is shown in Fig. 2d (also shown in Additional file 4: Table S3, Additional file 5: Table S4). Note that iCluster and intNMF are NMF-based methods and they do not generate similarity matrix, so *Dunn* index for the two methods is not provided. For the *Dunn* index, RWRF and RWRNF are both better than the concatenation, COCA, SNF and ANF. For the *P* value, Fig. 2d shows that RWRF and RWRNF are both better than the concatenation, COCA and the two joint dimensionality reduction methods except that RWRF performs worse than intNMF on one data set. As for the two similarity-based methods, Fig. 2d shows that RWRNF is better that them on five datasets, RWRF is superior to SNF on three datasets and to ANF on three datasets.

### Model analysis

#### Parameter selection

We use ACC data as an example to test the sensitivity of hyper-parameters of RWRF and RWRNF. On other data sets, parameter selection also has the similar results.

For RWRF and RWRNF, we varied γ and fixed other hyper-parameters, then *P* values for log-rank test and *Dunn* values are recorded (Additional file 6: Fig. S1). Here, *γ* = 0.6–0.9 is recommended. In this study, we choose *γ* = 0.7.

For RWRNF, *P* values for the log-rank test and *Dunn* values are recorded when *m*, *α*, and *β* are varied respectively (Additional file 7: Fig. S2). Here, *m* = 5–15 (In this study, we choose *m* = 10), *α* = 0.9, *β* = 0.9 are recommended.

#### Anti-noise ability

Simulated data is created (as in Methods section) to verify the anti-noise ability of RWRF and RWRNF.

**Fig. 3:**
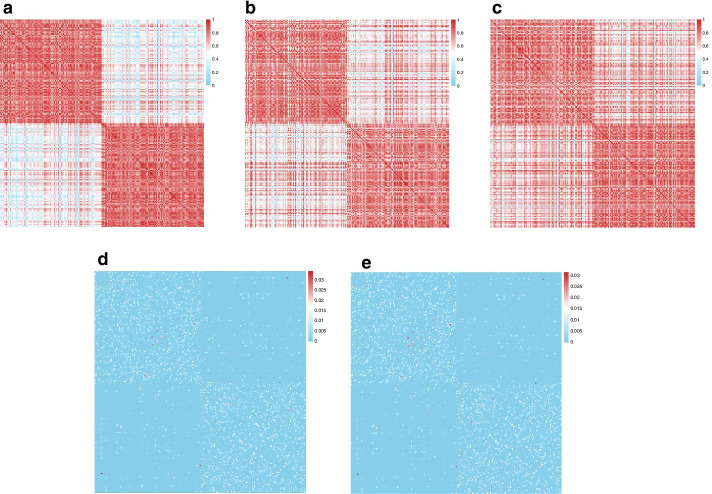
A simulation that verifies the anti-noise ability of RWRF and RWRNF. **a**–**c** The similarity heatmaps of the original data, the data with Gaussian noise and the data with Gamma noise. **d** The integrated network by using RWRF. **e** The integrated network by using RWRNF

To show the anti-noise ability more clearly, we drew similarity heatmap for each type of data. The similarity heatmap of the original data is shown in Fig. 3a. And the similarity heatmaps of the data with Gaussian noise and the data with Gamma noise are shown in Fig. 3b, c respectively. Comparing the heatmap of the original data and that of each noise addition data, we found that the original data has a clearer cluster structure than any of the noise addition data. Subsequently, we fused the data with Gaussian noise and the data with Gamma noise by using RWRF. The integrated similarity heatmap are shown in Fig. 3d. We also used RWRNF to fuse data with two noise (Fig. 3e). In the initial two matrices of noise addition data, there are many similarities between the two classes due to noise. But after the fusion, the between-class similarity is reduced and the within-class similarity is strengthened. To prove that, we performed spectral clustering and calculated normalized mutual information (NMI), which reflects consistency across the original true label and the results from the clustering procedure. The NMI indexes of the data with Gaussian noise and the data with Gamma noise are 0.60 and 0.56 respectively. After the fusion by RWRF and RWRNF, the NMI indexes are both 0.76. The results indicate that RWRF and RWRNF both have strong anti-noise ability.

For the data with Gaussian noise and the data with Gamma noise, we set standard deviation, shape parameter *α* and rate parameter *β* as 1.5, 3 and 1 respectively. Here, the parameters of the simulation are set as an example. In order to explore the influence of noise parameters on anti-noise performance, we conducted the following analysis.

NMI between cluster labels obtained by spectral clustering of the RWRF (and RWRNF) fused similarity matrix and the simulated ground truth plotted as a function of standard deviation, parameter *α* and parameter *β* respectively (Additional file 8: Fig. S3). Higher NMI corresponds to higher concordance between obtained clusters and ground truth. Increases in standard deviation, *α* and *β* will cause an increase in noise. RWRF and RWRNF maintain the anti-noise ability when the noise gradually increases.

For the artificial multi-omics datasets, we fused the three types of data using RWRF and RWRNF. Then spectral clustering is used on the similarity network for subtyping. The similarity heatmaps of the three types of data are shown in Additional file 9: Fig. S4b–d respectively. The integrated similarity heatmaps are shown in Additional file 9: Fig. S4e, f. After the fusion, the between-class similarity is reduced and the within-class similarity is strengthened. The NMI indexes of the simulated methylation data, the simulated gene expression data and the simulated protein expression data are 0.10, 0.27 and 0.58 respectively. After the fusion by RWRF and RWRNF, the NMI indexes are both 0.89. The results also show the strong anti-noise ability of RWRF and RWRNF.

### Case study

Here, we take the identification of adrenocortical carcinoma (ACC) molecular subtypes as an example to demonstrate the effectiveness of RWRF and RWRNF algorithms. Three types of omics data (mRNA expression, DNA methylation, and miRNA expression) are integrated as a sample similarity network. After that, molecular subtyping is conducted on the integrated network.

**Fig. 4:**
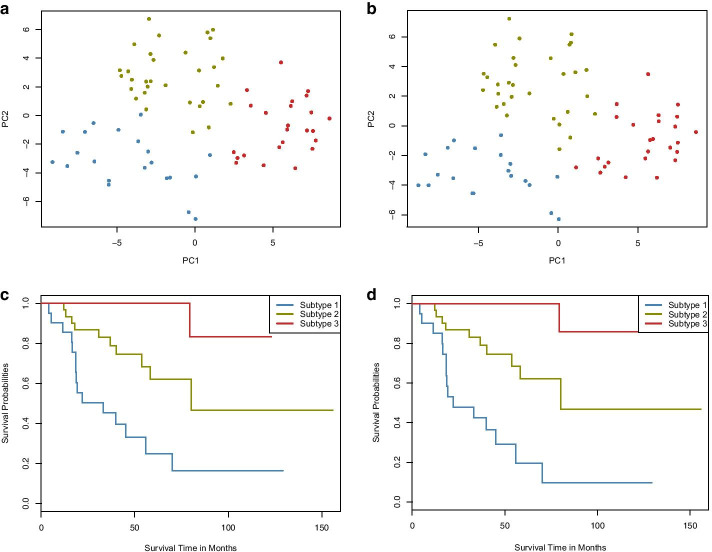
Effectiveness of molecular subtyping by using RWRF and RWRNF. **a**–**b** Through the first two principal components, samples colored by their cluster labels are plotted. **c**–**d** Kaplan–Meier survival curves for three ACC subtypes as identified from integrated data

PCA dimensionality reduction is then applied to the resulting integrated similarity network. Figure 4a, b (represent the results of RWRF and RWRNF respectively) show that samples colored by their cluster labels can be separated through the first two principal components. Kaplan–Meier survival curves are demonstrated in Fig. 4c, d (represent the results of RWRF and RWRNF respectively). In this research, we used overall survival (OS) in the survival analysis. OS is an important endpoint, with the advantage that there is minimal ambiguity in defining an OS event; the patient is either alive or dead [33]. Among three subtypes of ACC, subtype 1 results in the poorest overall survival.

**Fig. 5:**
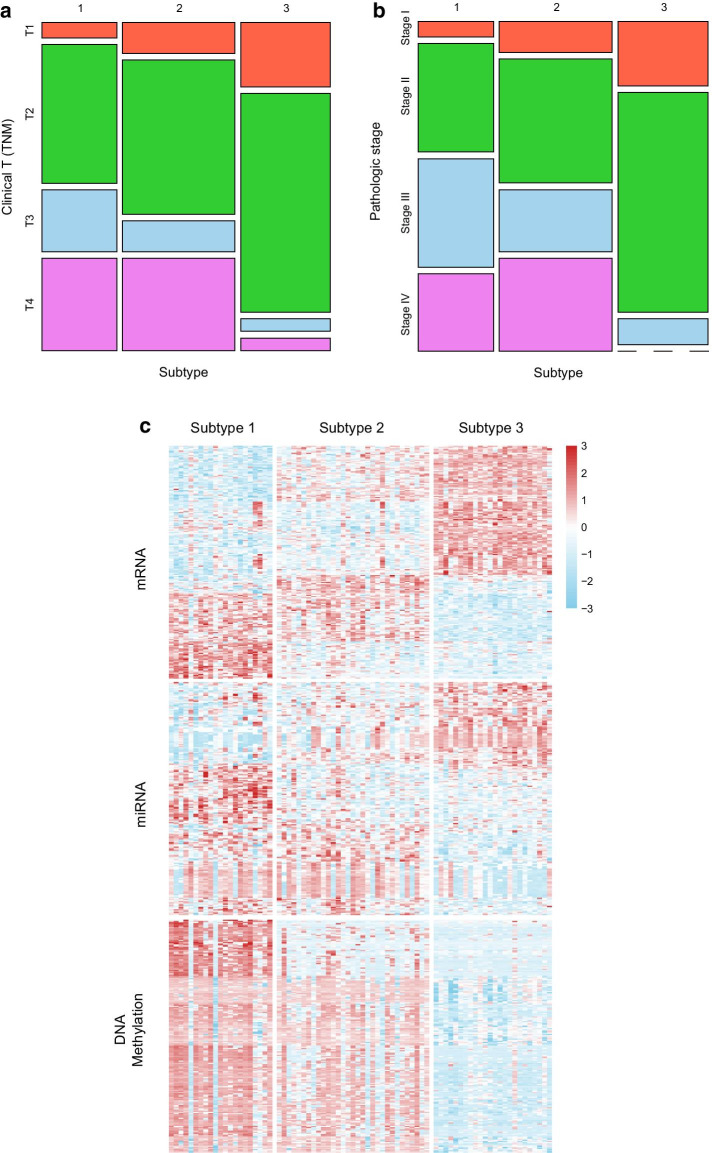
Clinical association of molecular subtype groups. **a** Distribution of tumor size across three subtype groups. **b** Distribution of stage at diagnosis across three subtype groups. **c** Heatmap of features significantly differential among ACC subtypes that are identified by using RWRF

We also found associations between the subtypes and clinical variables. The Clinical T (TNM) is based on the TNM staging system. It indicates the extent of the tumor (T). For the Clinical T (TNM) stage, the larger the number is, the worse the prognosis is. The pathologic stage combines the results of both the clinical staging (physical exam, imaging test) with surgical results. It estimates the extent of the cancer, where stage IV is the most serious condition. Tumors in subtype 3 are less aggressive considering the extent of the tumor (T) whereas tumors in subtype 1 tend to be diagnosed at more advanced stages (III and IV) (Fig. 5a, b). Furthermore, each of omics data has a distinct molecular pattern for 3 different molecular subtypes. Figure 5c shows the heatmap of features among the 3 ACC subtypes. We select and demonstrate these features whose NMI values are ranked in the top two hundred of all NMI values across all feature types (see Methods). Each subtype has a very different profile in mRNA expression, miRNA expression, and DNA methylation. And the signature difference between subtype 1 and subtype 3 is very obvious. The results indicate that these three subtypes may have different molecular mechanisms.

To illustrate the reliability of the different molecular features, we studied the biological contextualization in several cases.

Among the top two hundred important features of mRNA expression, gene G0S2, which significantly effects the prognosis of ACC patients (Additional file 10: Fig. S5), shows potential as a biomarker. In our findings, the expression of G0S2 in subtype 1 is the lowest, but the expression in subtype3 is the highest. Then we focus on the CpG islands of G0S2 in DNA methylation data. Except cg23646375 and cg07434244, nine CpG sites (cg02638691, cg06616057, cg08158408, cg08185241, cg09666230, cg14824901, cg17710021, cg26050864 and cg27176828) are among the top 5% important features of DNA methylation. The methylation levels at the nine CpG sites are also significantly different between subtype 1 and subtype 3 (Additional file 11: Fig. S6). This suggests that G0S2 methylation may be one of the biological characteristics of the subtype 1. Indeed, a recent study confirmed that hypermethylation of the G0S2 locus and decreased G0S2 expression are hallmarks of rapidly recurrent or fatal ACC [34]. Moreover, G0S2 hypermethylation and silencing is exclusive to ACC. G0S2 may have important roles in adrenocortical biology and is worthy of future investigation.

The contribution of deregulated miRNAs to the pathogenesis of ACC has been studied in recent years, and some miRNAs have been shown to carry potential diagnostic and prognostic values. Among the top important features of miRNA expression in this study, four miRNAs (miR-125b-5p, miR-139-3p, miR-139-5p and miR-335-5p) are shown as potential biomarkers in ACC according to previous researches [35–39]. Overexpressed miR-139-5p, and underexpressed miR-125b-5p, miR-139-3p and miR-335-5p were found associated with ACC aggressiveness and poor prognosis. Therefore, these four miRNAs may serve as diagnostic and prognostic markers.

Moreover, in order to show the difference in biological pathways between subtype 1 and subtype 3, we performed Kyoto Encyclopedia of Genes and Genomes (KEGG) and Gene Ontology Molecular Function (GOMF) enrichment analysis based on feature genes of subtype 1 and subtype 3 respectively (Additional file 12: Fig. S7). KEGG and GOMF enrichment results were selected by a *P* value threshold of 0.05. Compare to subtype 3, subtype 1 were significantly enriched in cortisol synthesis and secretion KEGG pathway. One of the symptoms of ACC is excessive levels of cortisol. Subtype 1, which has a poorer prognosis, may cause even more abnormal increases in cortisol levels. Furthermore, compare to subtype 3, subtype 1 were significantly enriched in methylation-related molecular function (histone binding, methylated histone binding and methylation-dependent protein binding). A recent study has shown that the genomes of rapidly recurrent carcinomas are characterized by aberrant methylation directed to promoter CpG islands [34].

## Discussion

Here, we proposed RWRF and RWRNF, two network-based methods for integrating multi-omics data. Appling RWRF and RWRNF to TCGA data, we integrated mRNA expression, miRNA expression, and DNA methylation data to identify cancer subtypes. Notably, RWRF and RWRNF both have strong anti-noise ability. Through comparing with single data type analysis and previous integrative methods, it is proven that RWRF and RWRNF are more effective.

The network fusion of RWRF and RWRNF goes beyond a simple integration representing an average or a maximum of sample similarity measurements. By the random walk with restart on multiplex network, RWRF and RWRNF can capture potential links between samples with high sensitivity. If the similarity score in a single network is high but the similarity score in other single networks are low, the methods do not lost the original information. The random walk process helps RWRF and RWRNF make full use of topology information of each similarity network, which contains each type of data’s information.

As highly extensible methods, RWRF and RWRNF can fuse various omics data beyond the three examined here. For example, metabolomics data and proteomics data can also be integrated by using our methods. Furthermore, besides cancer subtyping, RWRF and RWRNF have many other applications. In drug repositioning, integrating various drug properties could offer new insight into the universality and complementarity of drug properties.

Nevertheless, both RWRF and RWRNF have their limitations. Multi-omics data are frequently profiled from different sets of patients/samples, leading to missing data. One of the limitations of this research is the limited set of samples using the intersection of different sample sets with multi-dimensional features. Data with more than 20% of missing values were removed and remaining missing values were imputed. Missing values for mRNA expression, DNA methylation and miRNA expression were substituted by their mean value over all the cohorts.

In addition to imputation, there are some other approaches that deal with missing values. Similarity measurements that can tolerate missing values is a promising method. Sitaram et al. present an approach which can make use of the characteristics of the mahalanobis distance to inherently accommodate the missing values [40]. Li et al. proposed a matrix calibration approach, which can estimating jaccard index with missing observations [41]. But these methods can only be applied to specific types of data or small data sets.

In general, with the accumulation of multi-omics data in the future, the scale of sample set will be expanded, and with the development of similarity measurements managing missing data, the performance of RWRF and RWRNF will be improved and the application scenarios will be expanded.

## Conclusions

In this study, we proposed two multi-omics data integration algorithms based on random walk with restart (RWR) on multiplex network, namely Random Walk with Restart for multi-dimensional data Fusion (RWRF) and Random Walk with Restart and Neighbor information-based multi-dimensional data Fusion (RWRNF) respectively. RWRF and RWRNF apply the theory of random walk to data integration and implement the integrated analysis of multi-omics data. By being used in cancer subtyping, the methods facilitate our understanding of cancer and precision treatment in clinical practice, and their high expansibility allows us to explore cancer from a wider range of fields.

## Methods

### RWRF algorithm

In this study, a multiplex network is a collection of *L* similarity networks, considered as layers, sharing the same set of *n* nodes [42, 43]. Here, suppose $L=2$, then each layer is defined by its $n\times n$ adjacency matrix, ${S}_{1}$ and ${S}_{2}$. They are constructed based on different features for the same batch of sample nodes. There are *n* samples in each of the networks. The identical samples in the two networks are connected to construct a multiplex network $S$:

4 $$S = \left[ {\begin{array}{*{20}c} {S_{1} } & {I_{n} } \\ {I_{n} } & {S_{2} } \\ \end{array} } \right]$$

where ${I}_{n}$ is diagonal matrix, indicating the connection between ${S}_{1}$ and ${S}_{2}$.

The whole algorithm consists of three steps: (1) initial value determination, (2) random walk with restart (RWR) on the multiplex network, (3) similarity network fusion.

First of all, we set the initial value of the algorithm.

If we want to obtain similarity scores between the *x*-th node ${C}_{x}^{(1)}$ in similarity network ${S}_{1}$ and other nodes, ${C}_{x}^{(1)}$ is denoted as the seed node in the multiplex network $S$. An imaginary particle starts a random walk from the seed node. In the similarity network ${S}_{1}$, probability 1 is assigned to the seed node ${C}_{x}^{(1)}$ and probability 0 is assigned to other nodes, forming the initial probability distribution ${\overrightarrow{u}}_{0}$ for similarity network ${S}_{1}$. Similarly, the initial probability distribution for the *x*-th node ${C}_{x}^{(2)}$ on similarity network ${S}_{2}$ is ${\overrightarrow{v}}_{0}$. Both ${\overrightarrow{u}}_{0}$ and ${\overrightarrow{v}}_{0}$ are n-dimensional vectors and satisfy the following conditions:

5 $$\sum\nolimits_{n} {\left| {\overrightarrow {u}_{0} } \right|} = 1$$

6 $$\sum\nolimits_{n} {\left| {\overrightarrow {v}_{0} } \right|} = 1$$

So, the initial probability distribution for the multiplex network $S$ is:

7 $$\overrightarrow {p}_{0} = \left[ {\begin{array}{*{20}c} {\alpha_{{1}} \overrightarrow {u}_{0} } \\ {\alpha_{{2}} \overrightarrow {v}_{0} } \\ \end{array} } \right]$$

8 $$\alpha_{1} + \alpha_{2} = 1$$

The parameters $\alpha_{{1}}$ and $\alpha_{{2}}$ ∈ [0, 1] weight the importance of similarity network $S_{1}$ and similarity network $S_{2}$. If there are *k* similarity networks, the first similarity network $S_{1}$ has the weight $\alpha$ and each of the other similarity networks has the weight $\frac{1 - \alpha }{{k - 1}}$.

After that, we implemented RWR on the multiplex network. RWR equation can be defined as:

9 $$\overrightarrow {p}_{t + 1} = \left( {1 - \gamma } \right)W^{T} \overrightarrow {p}_{t} + \gamma \overrightarrow {p}_{0}$$

where $\vec{p}_{0}$ is the initial probability distribution, $\vec{p}_{t}$ is a vector in which the *i*-th element indicates the probability for the situation that the imaginary particle walks to node *i* at step t. The parameter $\gamma$ is restart probability, which allows the restart of the random walk from the seed node at each step. This formula is iteratively updated. $W^{T}$ denotes a transition matrix defined as follows:

10 $$W = \left[ {\begin{array}{*{20}c} {W_{11} } & {W_{12} } \\ {W_{21} } & {W_{22} } \\ \end{array} } \right]$$

where $W_{11}$ and $W_{22}$ are intra-transition matrixes indicating the probability of walking from one node to other nodes in the same similarity network; ${ }W_{12}$ and $W_{21}$ are inter-transition matrixes. $W_{12}$ is the transition matrix indicating the probability of walking from the first similarity network $S_{1}$ to the second similarity network $S_{2}$, and $W_{21}$ is the transition matrix indicating the probability of walking from $S_{2}$ to $S_{1}$. Each entry $w_{{{\text{ij}}}}$ in the transition probability matrix W is the probability of a transition from node *j* to node *i*.

Suppose that in the process of random walk, the probability of walking from one node to other nodes in the same similarity network is $\lambda_{{1}}$, and the probability of jumping from one similarity network to other similarity networks is $\lambda_{{2}}$. $\lambda_{{1}}$ and $\lambda_{{2}}$ satisfy $\lambda_{1} + \lambda_{2} = 1$. The parameter $\lambda_{{1}}$ and $\lambda_{{2}}$ quantifies the probability of staying in a layer or jumping between the layers.

The transition matrix is defined as follows:

11 $$W_{11} \left( {i,j} \right) = \frac{{\lambda_{{1}} S_{1} \left( {i,j} \right)}}{{\sum\nolimits_{l} {S_{1} \left( {i,l} \right)} }}$$

12 $$W_{12} \left( {i,j} \right) = \left\{ \begin{gathered} \lambda_{{2}} ,\;\;\;\;\;if\;i = j \hfill \\ 0,\;\;\;\;\;\;otherwise \hfill \\ \end{gathered} \right.$$

where $W_{11} \left( {i,j} \right)$ is transition probability from node $C_{i}^{\left( 1 \right)}$ to $C_{j}^{\left( 1 \right)}$, $W_{12} \left( {i,j} \right)$ is transition probability from node $C_{i}^{\left( 1 \right)}$ to $C_{j}^{\left( 2 \right)}$. $S_{1} \left( {i,j} \right)$ indicates the similarity score between node $C_{i}^{\left( 1 \right)}$ and node $C_{j}^{\left( 1 \right)}$ in similarity network $S_{1}$. $\mathop \sum \limits_{l} S_{1} \left( {i,l} \right)$ represents the sum of the similarity scores between node $C_{i}^{\left( 1 \right)}$ and each of all other nodes in the similarity network $S_{1}$. $W_{22}$ and $W_{21}$ are defined similarly.

Iterations are repeated until the difference between $\vec{p}_{t}$ and $\vec{p}_{t + 1}$ falls below $10^{ - 10}$, as in previous studies [44, 45]. After several iterations, the stationary probability distribution $$\vec{p}_{stable} = \left[ {\begin{array}{*{20}c} {\vec{u}_{stable} } \\ {\vec{v}_{stable} } \\ \end{array} } \right]$$ will be obtained. The stationary probability distribution $\vec{p}_{stable}$ is usually reached after 16 iterations (Additional file 13: Fig. S8). Here, ${ }\vec{u}_{stable}$ is similarity scores between $C_{x}^{\left( 1 \right)}$ and other nodes in similarity network $S_{1}$, and $\vec{v}_{stable}$ is similarity scores between $C_{x}^{\left( 1 \right)}$ and other nodes in similarity network $S_{2}$.

When the stationary probability distribution is reached, the elements in $\vec{p}_{stable}$ represent a proximity measure from every graph node to the seed node. Considering that there are $2 \times n$ nodes in the multiplex network $S$, the above process was repeated $2 \times n$ times (the $2 \times n$ nodes were set as seeds respectively). Then we can obtain $2 \times n$ $\vec{p}_{stable}$ for $2 \times n$ seeds. The $2 \times n$ $\vec{p}_{stable}$ is combined to form a new multiplex network:

13 $$S^{^{\prime}} = \left[ {\begin{array}{*{20}c} {S_{1}^{^{\prime}} } & {A_{12}^{^{\prime}} } \\ {A_{21}^{^{\prime}} } & {S_{2}^{^{\prime}} } \\ \end{array} } \right]$$

$S_{1}^{^{\prime}}$ and $S_{2}^{^{\prime}}$ represent new similarity matrixes for each layer, which measure proximities among the nodes in each layer. $A_{12}^{^{\prime}}$ and $A_{21}^{^{\prime}}$ represent similarities between different layers.

The integrated similarity network is defined as follows:

14 $$S_{fusion} = \frac{{S_{1}^{^{\prime}} + S_{2}^{^{\prime}} + S_{1}^{^{\prime}T} + S_{2}^{^{\prime}T} }}{4}$$

Here, we set $\gamma = 0.7$. The value of the restart parameter will be kept in the following version of the network fusion algorithm. If there are *k* similarity networks, $\lambda_{1} = \lambda_{2} = \cdots = \lambda_{k} = 1/k,\,\alpha_{1} = \alpha_{2} = \cdots = \alpha_{k} = 1/k$.

### RWRNF algorithm

RWRNF algorithm is an extension of the RWRF algorithm. RWRNF algorithm considers not only the connected edges between the identical samples of different similarity networks, but also the neighborhood information of the samples in each similarity network.

Similarly, suppose $L = 2$, then each layer is defined by its $n \times n$ adjacency matrix, $S_{1}$ and $S_{2}$. They are constructed based on different features for the same batch of samples. There are *n* samples in each of the networks. ${ }C_{x}^{\left( 1 \right)}$ is the *x*-th node in similarity network $S_{1}$. $C_{x}^{\left( 2 \right)}$, the mirror node of $C_{x}^{\left( 1 \right)}$, is the *x*-th node in similarity network $S_{2}$.

We connect the nodes in the two similarity networks in the form of weighted directed edges as follows:

The m nearest neighbors of $C_{x}^{\left( 1 \right)}$ in similarity network $S_{1}$ is $\left\{ {C_{x1}^{\left( 1 \right)} ,C_{x2}^{\left( 1 \right)} , \ldots ,C_{xm}^{\left( 1 \right)} } \right\}$. The mirror nodes of this m nodes in similarity network $S_{2}$ is $\left\{ {C_{x1}^{\left( 2 \right)} ,C_{x2}^{\left( 2 \right)} , \ldots ,C_{xm}^{\left( 2 \right)} } \right\}$. $C_{x}^{\left( 1 \right)}$ and $C_{x}^{\left( 2 \right)}$ are connected by an directed edge pointing from $C_{x}^{\left( 1 \right)}$ to $C_{x}^{\left( 2 \right)}$ with the weight of $\beta$. $C_{x}^{\left( 1 \right)}$ (source node) and $\left\{ {C_{x1}^{\left( 2 \right)} ,C_{x2}^{\left( 2 \right)} , \ldots ,C_{xm}^{\left( 2 \right)} } \right\}$ (target nodes) are connected by directed edges where the weight of each edge is $\left( {1 - \beta } \right)/m$. Similarly, the m nearest neighbors of $C_{x}^{\left( 2 \right)}$ in similarity network $S_{2}$ is $\left\{ {C_{{x1^{\prime}}}^{\left( 2 \right)} ,C_{{x2^{\prime}}}^{\left( 2 \right)} , \ldots ,C_{{xm^{\prime}}}^{\left( 2 \right)} } \right\}$. The mirror nodes of this m nodes in similarity network $S_{1}$ is $\left\{ {C_{{x1^{\prime}}}^{\left( 1 \right)} ,C_{{x2^{\prime}}}^{\left( 1 \right)} , \ldots ,C_{{xm^{\prime}}}^{\left( 1 \right)} } \right\}$. $C_{x}^{\left( 2 \right)}$ and $C_{x}^{\left( 1 \right)}$ are connected by a directed edge pointing from $C_{x}^{\left( 2 \right)}$ to $C_{x}^{\left( 1 \right)}$ with the weight of $\beta$. $C_{x}^{\left( 2 \right)}$ (source node) and $\left\{ {C_{{x1^{\prime}}}^{\left( 1 \right)} ,C_{{x2^{\prime}}}^{\left( 1 \right)} , \ldots ,C_{{xm^{\prime}}}^{\left( 1 \right)} } \right\}$ (target nodes) are connected by directed edges where the weight of each edge is $\left( {1 - \beta } \right)/m$. Note that $\left\{ {C_{x1}^{\left( 1 \right)} ,C_{x2}^{\left( 1 \right)} , \ldots ,C_{xm}^{\left( 1 \right)} } \right\}$ and $\left\{ {C_{{x1^{\prime}}}^{\left( 2 \right)} ,C_{{x2^{\prime}}}^{\left( 2 \right)} , \ldots ,C_{{xm^{\prime}}}^{\left( 2 \right)} } \right\}$ are not necessarily the same because the nearest neighbors of $C_{x}^{\left( 1 \right)}$ and $C_{x}^{\left( 2 \right)}$ may be different. Then a multiplex network $S$ is constructed as follows:

15 $$S = \left[ {\begin{array}{*{20}c} {S_{1} } & {A_{12} } \\ {A_{21} } & {S_{2} } \\ \end{array} } \right]$$

where $A_{12}$ indicates the connected matrix from $S_{1}$ to $S_{2}$, $A_{21}$ indicates the connected matrix from $S_{2}$ to $S_{1}$. $A_{12}$ and $A_{21}$ satisfy the following requirements:

16 $$\sum\nolimits_{m} {A_{12} \left( {i,m} \right) = 1}$$

17 $$\sum\nolimits_{m} {A_{21} \left( {i,m} \right) = 1}$$

First of all, we set the initial value of the algorithm.

If we want to obtain similarity scores between the *x*-th node $C_{x}^{\left( 1 \right)}$ in the similarity network $S_{1}$ and other nodes, $C_{x}^{\left( 1 \right)}$ is denoted as the seed node in the multiplex network $S$. An imaginary particle starts a random walk from the seed node. In the similarity network $S_{1}$, probability 1 is assigned to the seed node $C_{x}^{\left( 1 \right)}$ and probability 0 is assigned to other nodes, forming the initial probability distribution $\vec{u}_{0}$ for similarity network $S_{1}$. The initial probability distribution for similarity network $S_{2}$ is $\vec{v}_{0}$:

18 $$\overrightarrow {v}_{0} = A_{12} \left( {x,\;} \right)$$

where probability values are assigned to $C_{x}^{\left( 2 \right)}$ and $\left\{ {C_{x1}^{\left( 2 \right)} ,C_{x2}^{\left( 2 \right)} , \ldots ,C_{xm}^{\left( 2 \right)} } \right\}$ according to $A_{12} \left( x \right)$. Both $\vec{u}_{0}$ and $\vec{v}_{0}$ are n-dimensional vectors and satisfy the conditions of Eqs. (5) and (6).

So, the initial probability distribution for the multiplex network is:

19 $$\vec{p}_{0} = \left[ {\begin{array}{*{20}c} {\alpha \vec{u}_{0} } \\ {\left( {1 - \alpha } \right)\vec{v}_{0} } \\ \end{array} } \right]$$

The parameter $\alpha$ ∈ [0,1] weights the importance of similarity network $S_{1}$ and similarity network $S_{2}$. If there are *k* similarity networks, the first similarity network $S_{1}$ has the weight $\alpha$ and each of the other similarity networks has the weight $\frac{1 - \alpha }{{k - 1}}$.

After that, we implemented RWR on the multiplex network. RWR equation can be defined as:

20 $$\overrightarrow {p}_{t + 1} = \left( {1 - \gamma } \right)W^{T} \overrightarrow {p}_{t} + \gamma \overrightarrow {p}_{0}$$

where $\vec{p}_{0}$ is the initial probability distribution. $\vec{p}_{t}$ is a vector in which the *i*-th element indicates the probability for the situation that the imaginary particle walks to node *i* at step t. The parameter $\gamma$ is restart probability, which allows the restart of the random walk from the seed nodes at each step. This formula is iteratively updated. $W^{T}$ denotes a transition matrix defined as follows:

21 $$W = \left[ {\begin{array}{*{20}c} {W_{11} } & {W_{12} } \\ {W_{21} } & {W_{22} } \\ \end{array} } \right]$$

where $W_{11}$ and $W_{22}$ are intra-transition matrixes indicating the probability of walking from one node to other nodes in the same similarity network; $W_{12}$ and $W_{21}$ are inter-transition matrixes. $W_{12}$ is the transition matrix indicating the probability of walking from the first similarity network $S_{1}$ to the second similarity network $S_{2}$, and $W_{21}$ is the transition matrix indicating the probability of walking from $S_{2}$ to $S_{1}$. Each entry $w_{{{\text{ij}}}}$ in the transition probability matrix W is the probability of a transition from node *j* to node *i*.

Suppose that in the process of random walk, the probability of walking from one node to other node in the same similarity network is $\lambda_{{1}}$, and the probability of jumping from one similarity network to the other similarity network is $\lambda_{{2}}$. $\lambda_{{1}}$ and $\lambda_{{2}}$ satisfy $\lambda_{1} + \lambda_{2} = 1$. The parameter $\lambda_{{1}}$ and $\lambda_{{2}}$ quantifies the probability of staying in a layer or jumping between the layers.

The transition matrix is defined as follows:

22 $$W_{11} \left( {i,j} \right) = \frac{{\lambda_{1} S_{1} \left( {i,j} \right)}}{{\sum\nolimits_{l} {S_{1} \left( {i,l} \right)} }}$$

23 $$W_{12} \left( {i,j} \right) = \frac{{\lambda_{2} A_{12} \left( {i,j} \right)}}{{\sum\nolimits_{m} {A_{12} \left( {i,m} \right)} }}$$

where $W_{11} \left( {i,j} \right)$ is transition probability from node $C_{i}^{\left( 1 \right)}$ to $C_{j}^{\left( 1 \right)}$, $W_{12} \left( {i,j} \right)$ is transition probability from node $C_{i}^{\left( 1 \right)}$ to $C_{j}^{\left( 2 \right)}$. $S_{1} \left( {i,j} \right)$ indicates the similarity score between node $C_{i}^{\left( 1 \right)}$ and node $C_{j}^{\left( 1 \right)}$ in similarity network $S_{1}$. $\mathop \sum \limits_{l} S_{1} \left( {i,l} \right)$ represents the sum of the similarity scores between node $C_{i}^{\left( 1 \right)}$ and each of all other nodes in the similarity network $S_{1}$. $A_{12} \left( {i,j} \right)$ indicates the connection weight from node $C_{i}^{\left( 1 \right)}$ to node $C_{j}^{\left( 2 \right)}$. $\mathop \sum \limits_{m} A_{12} \left( {i,m} \right)$ represents the sum of the connection weights from node $C_{i}^{\left( 1 \right)}$ to each of $\left\{ {C_{x1}^{\left( 2 \right)} ,C_{x2}^{\left( 2 \right)} , \ldots ,C_{xm}^{\left( 2 \right)} } \right\}$ (The m nearest neighbors of $C_{x}^{\left( 1 \right)}$ in similarity network $S_{1}$ is $\left\{ {C_{x1}^{\left( 1 \right)} ,C_{x2}^{\left( 1 \right)} , \ldots ,C_{xm}^{\left( 1 \right)} } \right\}$, The mirror nodes of this m nodes in similarity network $S_{2}$ is $\left\{ {C_{x1}^{\left( 2 \right)} ,C_{x2}^{\left( 2 \right)} , \ldots ,C_{xm}^{\left( 2 \right)} } \right\}$). $W_{22}$ and $W_{21}$ are defined similarly.

Iterations are repeated until the difference between $\vec{p}_{t}$ and $\vec{p}_{t + 1}$ falls below $10^{ - 10}$, as in previous studies [44, 45]. After several iterations, the stationary probability distribution $$\vec{p}_{stable} = \left[ {\begin{array}{*{20}c} {\vec{u}_{stable} } \\ {\vec{v}_{stable} } \\ \end{array} } \right]$$ will be obtained. The stationary probability distribution $\vec{p}_{stable}$ is usually reached after 16 iterations (Additional file 13: Fig. S8). Here, $\vec{u}_{stable}$ is similarity scores between $C_{x}^{\left( 1 \right)}$ and other nodes in similarity network $S_{1}$, and $\vec{v}_{stable}$ is similarity scores between $C_{x}^{\left( 1 \right)}$ and other nodes in similarity network $S_{2}$.

When the stationary probability distribution is reached, the elements in $\vec{p}_{stable}$ represent a proximity measure from every graph node to the seed node. Considering that there are $2 \times n$ nodes in the multiplex network $S$, the above process was repeated $2 \times n$ times (the $2 \times n$ nodes were set as seeds respectively). Then we can obtain $2 \times n$ $\vec{p}_{stable}$ for $2 \times n$ seeds. The $2 \times n$ $\vec{p}_{stable}$ is combined to form a new multiplex network:

24 $$S^{^{\prime}} = \left[ {\begin{array}{*{20}c} {S_{1}^{^{\prime}} } & {A_{12}^{^{\prime}} } \\ {A_{21}^{^{\prime}} } & {S_{2}^{^{\prime}} } \\ \end{array} } \right]$$

$S_{1}^{^{\prime}}$ and $S_{2}^{^{\prime}}$ represent new similarity matrixes for each layer, which measure proximities among the nodes in each layer. $A_{12}^{^{\prime}}$ and $A_{21}^{^{\prime}}$ represent similarities between different layers.

The integrated similarity network is defined as follows:

25 $$S_{fusion} = \frac{{S_{1}^{^{\prime}} + S_{2}^{^{\prime}} + S_{1}^{^{\prime}T} + S_{2}^{^{\prime}T} }}{4}$$

Here, we set $m = 10$, $\alpha = 0.9$, $\beta = 0.9$, $\gamma = 0.7$. If there are *k* similarity networks, $\lambda_{1} = \lambda_{2} = \cdots = \lambda_{k} = 1/k$.

### Simulated data generation

To verify the anti-noise ability of RWRF and RWRNF, simulated data is generated. First, two clusters that are linearly separable are generated. There are 200 samples in the simulated data. The first 100 samples belong to one class, the second 100 samples belong to the other class. The original distribution of the simulated data is shown in Additional file 14: Fig. S9a. Two types of noise are added to the original data: (1) Gaussian noise with a mean of 0 and a standard deviation of 1.5 (Additional file 14: Fig. S9b); (2) Gamma noise with shape parameter *α* = 3 and rate parameter *β* = 1 (Additional file 14: Fig. S9c).

Moreover, we used artificial multi-omics datasets (Additional file 9: Fig. S4), which is closer to what is expected in real multi-omics dataset. We simulated these datasets using the “InterSIM” R package [46].

This package simulates multiple interrelated data types with realistic intra- and inter-relationships based on the DNA methylation, mRNA gene expression, and protein expression from the TCGA ovarian cancer study. InterSIM generates clusters and associates features to these clusters by shifting their mean values by a fixed amount.

In this study, we simulated multi-omics data with five clusters. The simulated data contains three types of data. There are 100 samples in each type of data. The simulated methylation data has 367-dimensional features, the simulated gene expression data has 131-dimensional features and the simulated protein expression data has 160-dimensional features.

### Feature selection

To select important features for each of the subtypes, we perform the following operations. For each of all features (including features in mRNA expression, DNA methylation and miRNA expression), a network is constructed independently. Then we use spectral clustering on the network to obtain subtypes. After that, we calculate NMI between the resulting cluster label and the RWRF (and RWRNF) found subtypes. The lager the NMI value is, the more likely the network based on the feature is to have the same subtypes as RWRF (and RWRNF) found subtypes. Therefore this feature is more important to the fused network structure. At last, according to the NMI value, we can select top ranking features, which are relatively important for the integrated network.

## Supplementary Information

**Additional file 1.** Cluster validity indexes.

**Additional file 2: Table S1.** *Dunn* index comparison before and after the fusion in six different cancer data set.

**Additional file 3: Table S2.** *P* value for log-rank test comparison before and after the fusion in six different cancer data set.

**Additional file 4: Table S3.** *Dunn* index comparison with concatenation, COCA, SNF and ANF in six different cancer data set.

**Additional file 5: Table S4.** *P* value for log-rank test comparison with Concatenation, COCA, iCluster, intNMF and two similarity-based methods: SNF and ANF in six different cancer data set.

**Additional file 6: Fig. S1.** Parameter selection for RWRF and RWRNF. **a** *P* values for log-rank test varies with parameter *γ*. **b** *Dunn* values varies with parameter *γ*.

**Additional file 7: Fig. S2.** Parameter selection for RWRNF. **a** *P* values for log-rank test varies with parameter *m*. **b** *Dunn* values varies with parameter *m*. **c** *P* values for log-rank test varies with parameter *α*. **d** *Dunn* values varies with parameter *α*. **e** *P* values for log-rank test varies with parameter *β*. **f** *Dunn* values varies with parameter *β*.

**Additional file 8: Fig. S3.** Anti-noise ability of RWRF and RWRNF. NMI between cluster labels obtained by spectral clustering of the RWRF (and RWRNF) fused similarity matrix and the simulated ground truth varies with parameter standard deviation of Gaussian noise (**a**), parameter α of Gamma noise (**b**) and parameter β of Gamma noise (**c**).

**Additional file 9: Fig. S4.** Artificial multi-omics datasets that verifies the anti-noise ability of RWRF and RWRNF. **a** The heatmaps of the generated methylation data, gene expression data and protein expression data. **b**, **c** The similarity heatmaps (after network clustering) of the methylation data, gene expression data and protein expression data. **d** The similarity heatmap (after network clustering) of the integrated network by using RWRF. **e** The similarity heatmap (after network clustering) of the integrated network by using RWRNF. The heatmaps of the methylation data, gene expression data and protein expression data are plotted by using “InterSIM” R package.

**Additional file 10: Fig. S5.** Survival analysis of the association between the expression levels of G0S2 and overall survival time in ACC. Kaplan–Meier survival curves for three ACC subtypes which is identified based on the expression levels of G0S2. Patients were classified in three different categories according to mRNA expression of gene G0S2.

**Additional file 11: Fig. S6.** The methylation levels at the CpG sites of G0S2. Heatmap of CpG sites’ methylation levels of G0S2 among ACC subtypes that are identified by using RWRF.

**Additional file 12: Fig. S7.** KEGG and GOMF enrichment analysis. **a**, **b** Dot plots of KEGG enrichment results of subtype1 annotation genes (**a**) and subtype3 annotation genes (**b**). **c**, **d** Dot plots of GOMF enrichment results of subtype1 annotation genes (**c**) and subtype3 annotation genes (**d**). Dot size corresponds to enriched gene quantity. Dot color corresponds to the significance of enrichment. Subtype1 annotation genes and subtype3 annotation genes are these mRNA genes that are significantly differential among ACC subtypes that are identified by using RWRF.

**Additional file 13: Fig. S8.** Algorithm convergence. The stationary probability distribution $\vec{p}_{stable}$ is usually reached after 16 iterations.

**Additional file 14: Fig. S9**. Distribution of the simulated data. **a** The distribution of the original data. **b** The distribution of the simulated data that are added Gaussian noise with a mean of 0 and a standard deviation of 1.5. **c** The distribution of the simulated data that are added Gamma noise with shape parameter *α* = 3 and rate parameter *β* = 1.

## Data Availability

The datasets generated and analyzed during the current study are available in The Cancer Genome Atlas (TCGA) repository, https:// portal.gdc.cancer.gov [1]. Moreover, source code is available at https://github.com/Sepstar/RWRF/.

## Abbreviations

RWR: Random walk with restart
RWRF: Random walk with restart for multi-dimensional data fusion
RWRNF: Random walk with restart and neighbor information-based multi-dimensional data fusion
TCGA: The cancer genome atlas
COCA: Cluster-of-cluster assignments
SNF: Similarity network fusion
ANF: Affinity network fusion
ACC: Adrenocortical carcinoma
BLCA: Bladder urothelial carcinoma
HNSC: Head and neck squamous cell carcinoma
UVM: Uveal melanoma
PAAD: Pancreatic adenocarcinoma
THCA: Thyroid carcinoma
NMF: Negative matrix factorization
NMI: Normalized mutual information
OS: Overall survival
KEGG: Kyoto encyclopedia of genes and genomes
GOMF: Gene ontology molecular function
