# Supplementary material for: Multi-dimensional data integration algorithm based on random walk with restart
Source: BMC Bioinformatics. 2021 Feb 27;22:97. doi: 10.1186/s12859-021-04029-3 (PMC7912853; doi:10.1186/s12859-021-04029-3)
Supplement: Supplementary file 1 — Additional file 1. Cluster validity indexes. [file 12859_2021_4029_MOESM1_ESM.pdf]

Cluster validity indexes

Ratkowsky Lance index

The cluster number is determined by seven cluster validity indexes: Ratkowsky Lance, Tau, Silhouette, C-index, SD-scat, SD-Dis and Calinski-Harabasz. These indexes are introduced as follows:

Ratkowsky Lance index is based on this formula:

$$\frac{\bar{S}}{q^{1/2}}$$

The value of $\bar{S}$ is equal to the average of the ratios of B/T where B stands for the sum of squares between the clusters for each variable and T for the total sum of squares for each variable.

The optimal number of clusters is that value of q for which $\frac{\bar{S}}{q^{1/2}}$ has its maximum value. If the value of q is made constant, the Ratkowsky Lance criterion can be reduced from $\frac{\bar{S}}{q^{1/2}}$ to $\bar{S}$.

Tau index

Tau index is computed as follows:

$$Tau = \frac{s(+) - s(-)}{[(n_d(n_d-1)/2 - t)(n_d(n_d-1)/2)]^{1/2}}$$

where $s(+)$ is the number of concordant comparisons. $s(-)$ is the number of discordant comparisons. n_d is the total number of distances (which is the same as the total number of observations or objects under study). t is the number of comparisons

of two pairs of points where both pairs represent within cluster comparisons or both pairs are between cluster comparisons.

Silhouette

The silhouette value is a measure of how well each object lies within its cluster. The silhouette value has a value between -1 and 1, and should be maximized. It is calculated as follows:

$$S(i) = \frac{b_i - a_i}{\max(b_i, a_i)}$$

where a_i is the average distance between sample i and all other data within the same cluster, b_i is the lowest average distance of sample i to all points in any other cluster.

C-index

C-index is measures of between-cluster isolation and within-cluster coherence. It can be defined as

$$cindex = \frac{D_u - (r \times D_{min})}{(r \times D_{max}) - (r \times D_{min})}$$

$$D_{min} \neq D_{max}$$

$cindex \in (0,1)$. D_u is the sum of all within-cluster dissimilarities. r is number of within-cluster dissimilarities. D_{min} smallest within-cluster dissimilarity. D_{max} largest within-cluster dissimilarity. The value of q (the number of clusters) which minimizes cindex is considered as specifying the number of clusters.

SD-scat

SD-scat is the average scattering of the clusters which is a measure of compactness of the clusters, defined as

$$Scat(q) = \frac{1}{q}\sum_{k=1}^{q} \|\sigma(c_k)\| / \|\sigma(X)\|$$

where q is the number of clusters, $\sigma(c_k)$ is the variance of cluster c_k, $\sigma(X)$ is the variance of data set X. $|X| = (X^T X)^2)^{1/2}$, where X is a vector. The number of clusters, q, that minimizes the index can be considered as an optimal value for the number of clusters present in the data set.

SD-Dis

SD-Dis evaluates the density of the area between the two clusters in relation to the density of the two clusters. Thus, it is a measure of the separation of the clusters, defined as

$$\mathrm{Dis}(q) = \frac{D_{max}}{D_{min}}\sum_{k=1}^{q}\left(\sum_{z=1}^{q} \|c_k - c_z\|\right)^{-1}$$

where $D_{max} = max(\|c_k - c_z\|)\ \forall k, z \in \{1,2,3, \dots, q\}$ is the maximum distance between cluster centers. $D_{min} = min(\|c_k - c_z\|)\ \forall k, z \in \{1,2,3, \dots, q\}$ is the minimum distance between cluster centers. The number of clusters, q, that minimizes the index can be considered as an optimal value for the number of clusters present in the data set.

Calinski-Harabasz index

The Calinski-Harabasz index

$$CH(q) = \frac{\mathrm{trace}(B_q)/(q-1)}{\mathrm{trace}(W_q)/(n-q)}$$

where $W_q = \sum_{k=1}^{q} \sum_{i \in C_k} (x_i - c_k)(x_i - c_k)^T$ is the within-group dispersion matrix for data clustered into q clusters. $B_q = \sum_{k=1}^{q} n_k * (c_k - c)(c_k - c)^T$ is the between-group dispersion matrix for data clustered into q clusters. x_i is p-dimensional vector of observations of the ith object in cluster k. c_k is centroid of cluster k. c is centroid of data matrix. n_k is number of objects in cluster C_k. The value of q, which minimizes $CH(q)$, is considered as specifying the number of clusters.
