# Supplementary material for: Multi-dimensional data integration algorithm based on random walk with restart
Source: BMC Bioinformatics. 2021 Feb 27;22:97. doi: 10.1186/s12859-021-04029-3 (PMC7912853; doi:10.1186/s12859-021-04029-3)
Supplement: Supplementary file 2 — Additional file 2: Table S1. Dunn index comparison before and after the fusion in six different cancer data set. [file 12859_2021_4029_MOESM2_ESM.docx]

**S5 Table. *Dunn* index comparison before and after the fusion in six different cancer data set.**

|  | **ACC** | **BLCA** | **HNSC** | **UVM** | **PAAD** | **THCA** |
| --- | --- | --- | --- | --- | --- | --- |
| **RWRF** | 0.745 | 0.627 | 0.591 | 0.700 | 0.651 | 0.533 |
| **RWRNF** | 0.723 | 0.595 | 0.589 | 0.682 | 0.639 | 0.534 |
| **mRNA** | 0.281 | 0.087 | 0.087 | 0.144 | 0.242 | 0.197 |
| **miRNA** | 0.147 | 0.037 | 0.058 | 0.161 | 0.222 | 0.268 |
| **Methylation** | 0.116 | 0.086 | 0.058 | 0.268 | 0.236 | 0.171 |
