# Supplementary material for: Multi-dimensional data integration algorithm based on random walk with restart
Source: BMC Bioinformatics. 2021 Feb 27;22:97. doi: 10.1186/s12859-021-04029-3 (PMC7912853; doi:10.1186/s12859-021-04029-3)
Supplement: Supplementary file 3 — Additional file 3: Table S2. P value for log-rank test comparison before and after the fusion in six different cancer data set. [file 12859_2021_4029_MOESM3_ESM.docx]

**S6 Table. *P* value for log-rank test comparison before and after the fusion in six different cancer data set.**

|  | **ACC** | **BLCA** | **HNSC** | **UVM** | **PAAD** | **THCA** |
| --- | --- | --- | --- | --- | --- | --- |
| **RWRF** | 5.08e-06 | 9.19e-03 | 4.10e-02 | 6.08e-09 | 1.04e-04 | 1.43e-02 |
| **RWRNF** | 1.84e-07 | 1.93e-03 | 6.23e-04 | 5.97e-09 | 2.17e-04 | 1.47e-02 |
| **mRNA** | 1.80e-04 | 3.35e-01 | 5.59e-01 | 3.92e-06 | 2.01e-03 | 6.55e-02 |
| **miRNA** | 1.37e-04 | 1.57e-01 | 4.35e-01 | 1.50e-08 | 4.38e-02 | 2.00e-01 |
| **Methylation** | 6.63e-04 | 3.58e-01 | 8.55e-01 | 3.27e-01 | 9.15e-03 | 1.13e-02 |
