# Supplementary material for: Multi-dimensional data integration algorithm based on random walk with restart
Source: BMC Bioinformatics. 2021 Feb 27;22:97. doi: 10.1186/s12859-021-04029-3 (PMC7912853; doi:10.1186/s12859-021-04029-3)
Supplement: Supplementary file 4 — Additional file 4: Table S3. Dunn index comparison with concatenation, COCA, SNF and ANF in six different cancer data set. [file 12859_2021_4029_MOESM4_ESM.docx]

**S7 Table. *Dunn* index comparison with Concatenation, COCA, SNF and ANF in six different cancer data set.**

|  | **ACC** | **BLCA** | **HNSC** | **UVM** | **PAAD** | **THCA** |
| --- | --- | --- | --- | --- | --- | --- |
| **RWRF** | 0.745 | 0.627 | 0.591 | 0.700 | 0.651 | 0.533 |
| **RWRNF** | 0.722 | 0.595 | 0.589 | 0.682 | 0.639 | 0.534 |
| **Concatenation** | 0.370 | 0.340 | 0.263 | 0.499 | 0.280 | 0.182 |
| **COCA** | 0.120 | 0.125 | 0.143 | 0.638 | 0.252 | 0.198 |
| **SNF** | 0.633 | 0.385 | 0.407 | 0.669 | 0.368 | 0.303 |
| **ANF** | 0.272 | 0.226 | 0.255 | 0.313 | 0.272 | 0.194 |
