# Supplementary material for: Multi-dimensional data integration algorithm based on random walk with restart
Source: BMC Bioinformatics. 2021 Feb 27;22:97. doi: 10.1186/s12859-021-04029-3 (PMC7912853; doi:10.1186/s12859-021-04029-3)
Supplement: Supplementary file 5 — Additional file 5: Table S4. P value for log-rank test comparison with Concatenation, COCA, iCluster, intNMF and two similarity-based methods: SNF and ANF in six different cancer data set. [file 12859_2021_4029_MOESM5_ESM.docx]

**S8 Table. *P* value for log-rank test comparison with Concatenation, COCA, iCluster, intNMF and two similarity-based methods: SNF and ANF in six different cancer data set.**

|  | **ACC** | **BLCA** | **HNSC** | **UVM** | **PAAD** | **THCA** |
| --- | --- | --- | --- | --- | --- | --- |
| **RWRF** | 5.08e-06 | 9.19e-03 | 4.10e-02 | 6.08e-09 | 1.04e-04 | 1.43e-02 |
| **RWRNF** | 1.84e-07 | 1.93e-03 | 6.23e-04 | 5.97e-09 | 2.17e-04 | 1.47e-02 |
| **Concatenation** | 9.32e-04 | 6.10e-02 | 6.60e-01 | 3.16e-06 | 8.98e-02 | 1.17e-01 |
| **COCA** | 2.16e-04 | 7.21e-02 | 1.18e-01 | 1.08e-04 | 4.00e-03 | 6.49e-02 |
| **iCluster** | 2.59e-03 | 7.06e-02 | 6.14e-02 | 2.39e-06 | 2.99e-03 | 2.33e-02 |
| **intNMF** | 3.36e-07 | 1.45e-01 | 9.91e-01 | 1.20e-07 | 1.37e-02 | 1.14e-01 |
| **SNF** | 2.33e-04 | 6.99e-03 | 1.75e-03 | 9.34e-08 | 2.07e-04 | 1.29e-02 |
| **ANF** | 1.29e-02 | 1.11e-01 | 6.68e-04 | 1.13e-06 | 5.84e-04 | 5.65e-03 |
