# Supplementary figures and images for: Multi-dimensional data integration algorithm based on random walk with restart
Source: BMC Bioinformatics. 2021 Feb 27;22:97. doi: 10.1186/s12859-021-04029-3 (PMC7912853; doi:10.1186/s12859-021-04029-3)

A

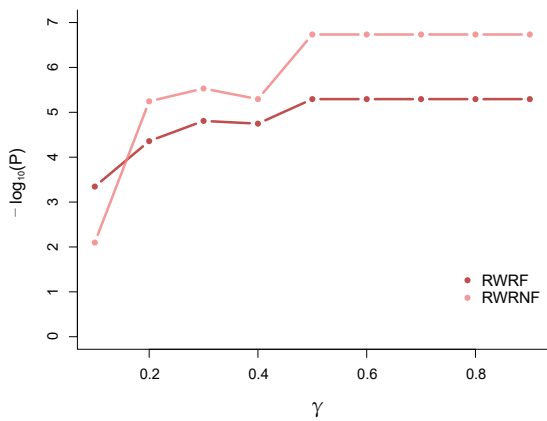

B

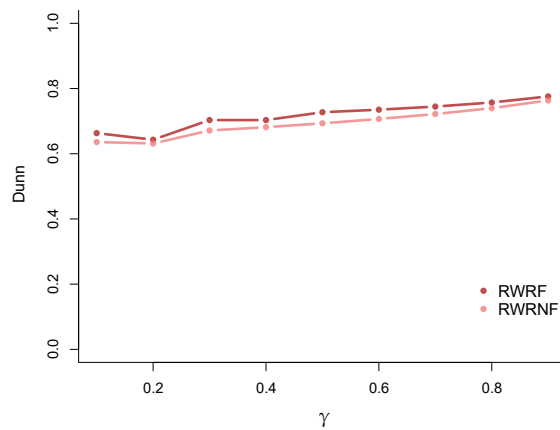

Supplement: Supplementary file 6 — Additional file 6: Fig. S1. Parameter selection for RWRF and RWRNF. a P values for log-rank test varies with parameter γ. b Dunn values varies with parameter γ. [file 12859_2021_4029_MOESM6_ESM.pdf]

A

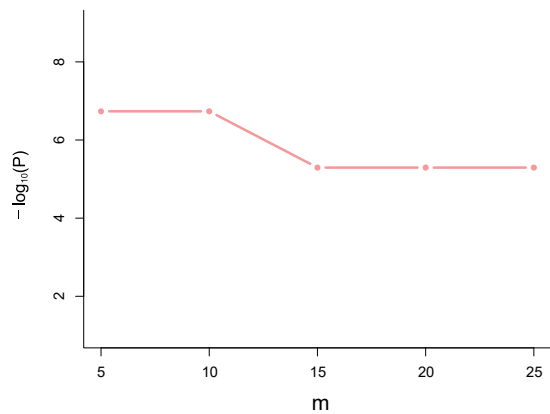

B

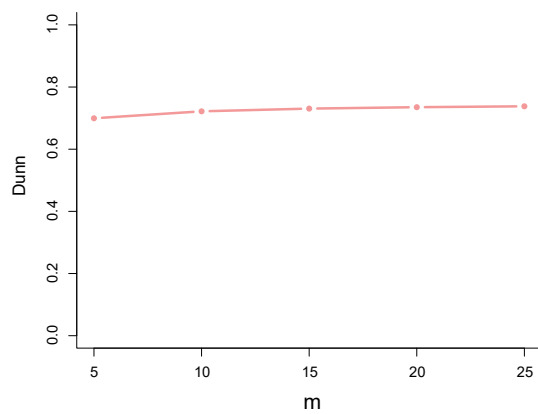

C

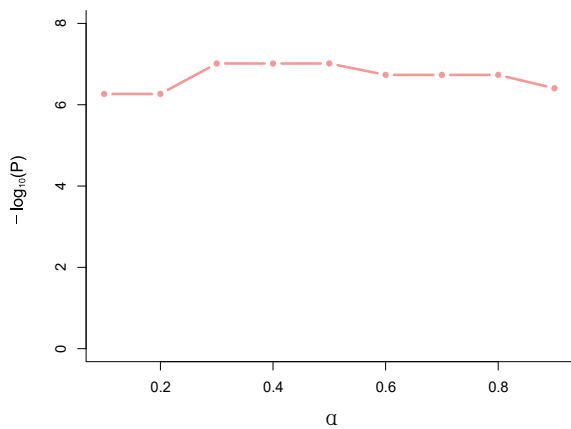

D

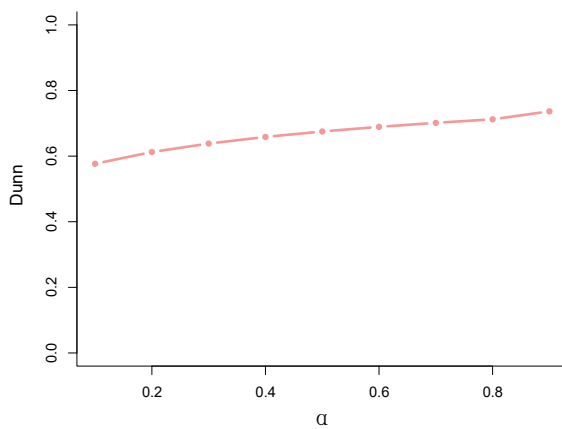

E

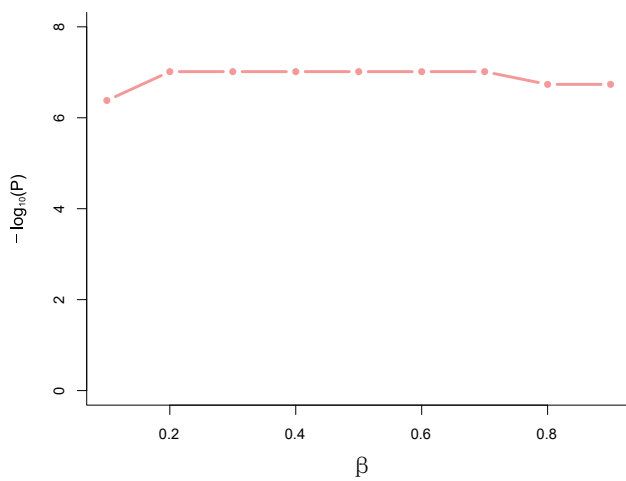

F

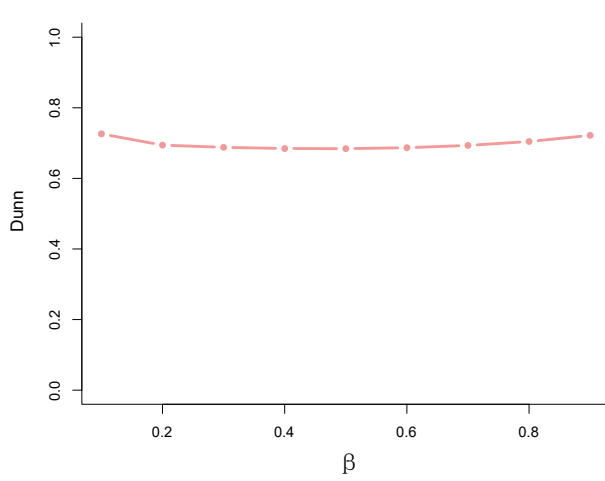

Supplement: Supplementary file 7 — Additional file 7: Fig. S2. Parameter selection for RWRNF. a P values for log-rank test varies with parameter m. b Dunn values varies with parameter m. c P values for log-rank test varies with parameter α. d Dunn values varies with parameter α. e P values for log-rank test varies with parameter β. f Dunn values varies with parameter β. [file 12859_2021_4029_MOESM7_ESM.pdf]

A

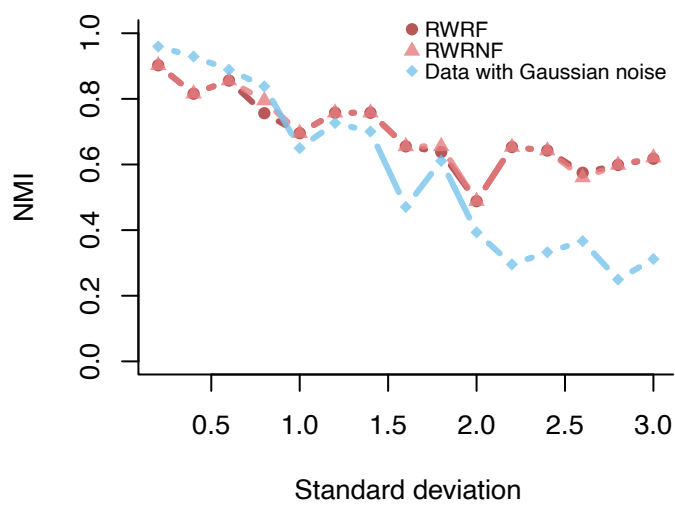

B

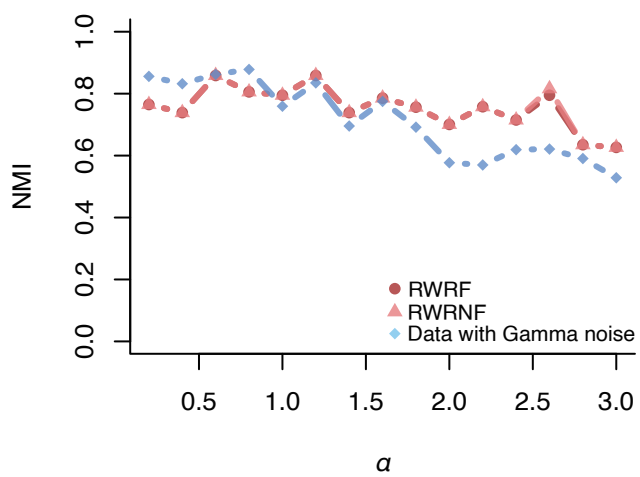

C

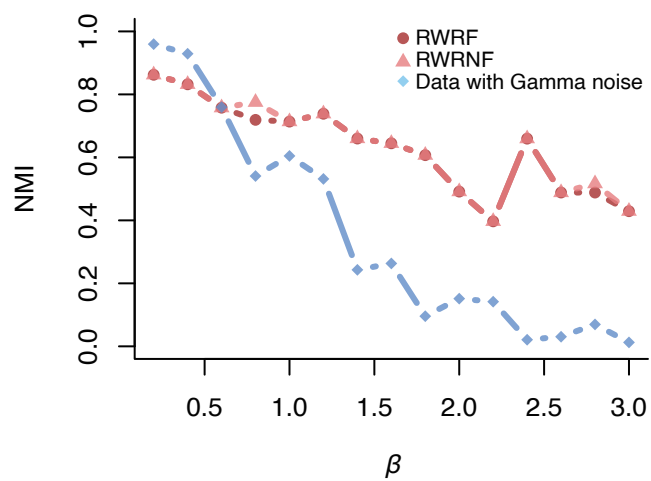

Supplement: Supplementary file 8 — Additional file 8: Fig. S3. Anti-noise ability of RWRF and RWRNF. NMI between cluster labels obtained by spectral clustering of the RWRF (and RWRNF) fused similarity matrix and the simulated ground truth varies with parameter standard deviation of Gaussian noise (a), parameter α of Gamma noise (b) and parameter β of Gamma noise (c). [file 12859_2021_4029_MOESM8_ESM.pdf]

A

Methylation

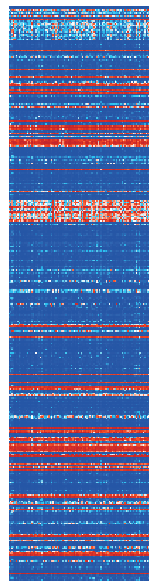

Gene expression

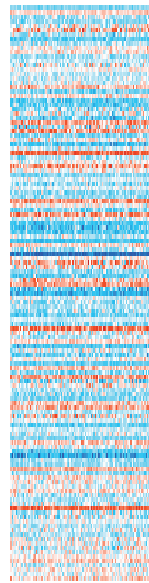

Protein expression

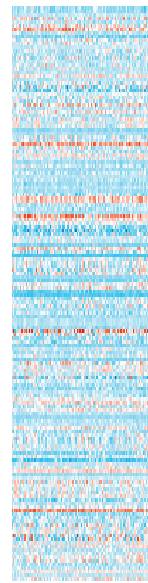

B

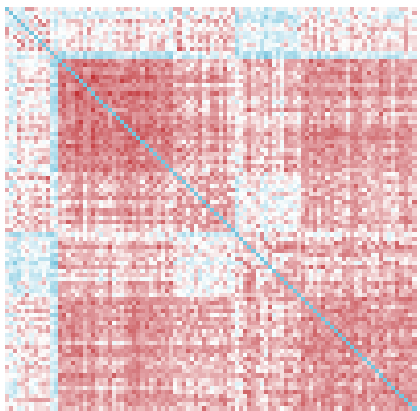

C

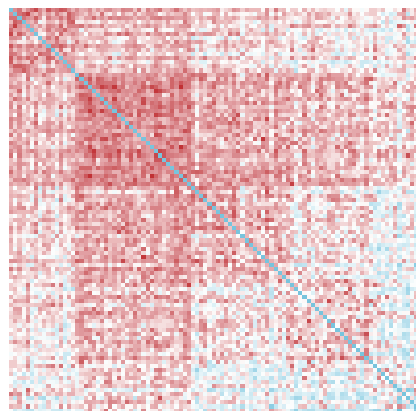

D

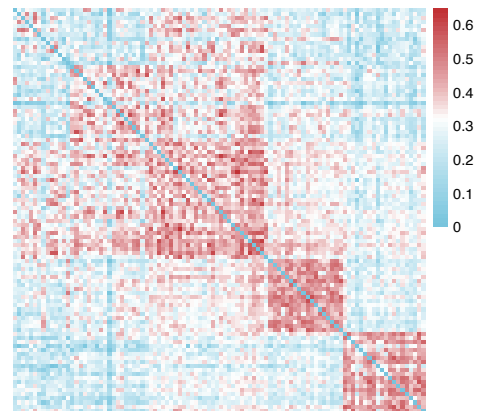

E

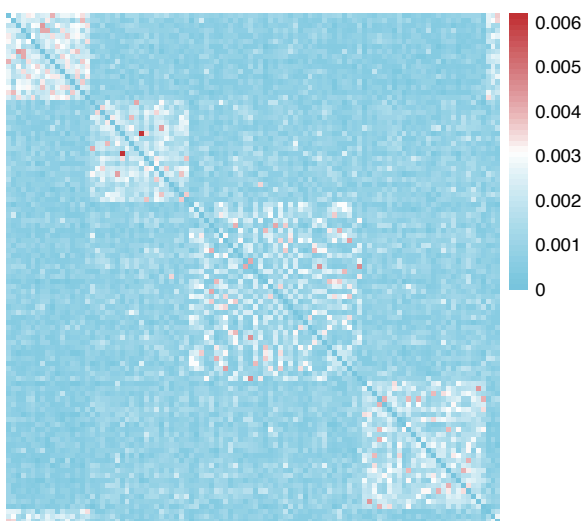

F

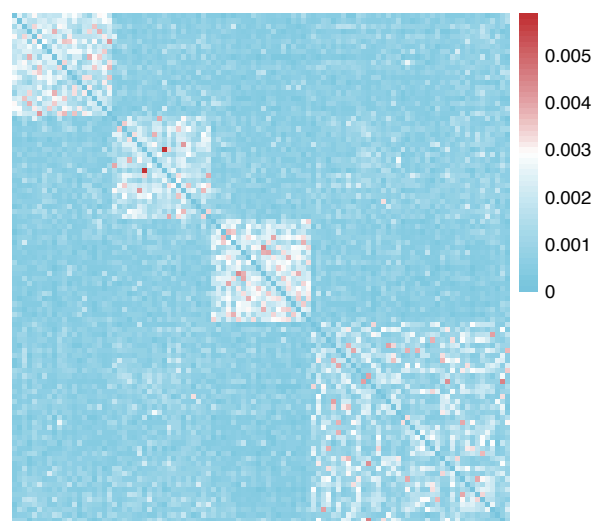

Supplement: Supplementary file 9 — Additional file 9: Fig. S4. Artificial multi-omics datasets that verifies the anti-noise ability of RWRF and RWRNF. a The heatmaps of the generated methylation data, gene expression data and protein expression data. b, c The similarity heatmaps (after network clustering) of the methylation data, gene expression data and protein expression data. d The similarity heatmap (after network clustering) of the integrated network by using RWRF. e The similarity heatmap (after network clustering) of the integrated network by using RWRNF. The heatmaps of the methylation data, gene expression data and protein expression data are plotted by using “InterSIM” R package. [file 12859_2021_4029_MOESM9_ESM.pdf]

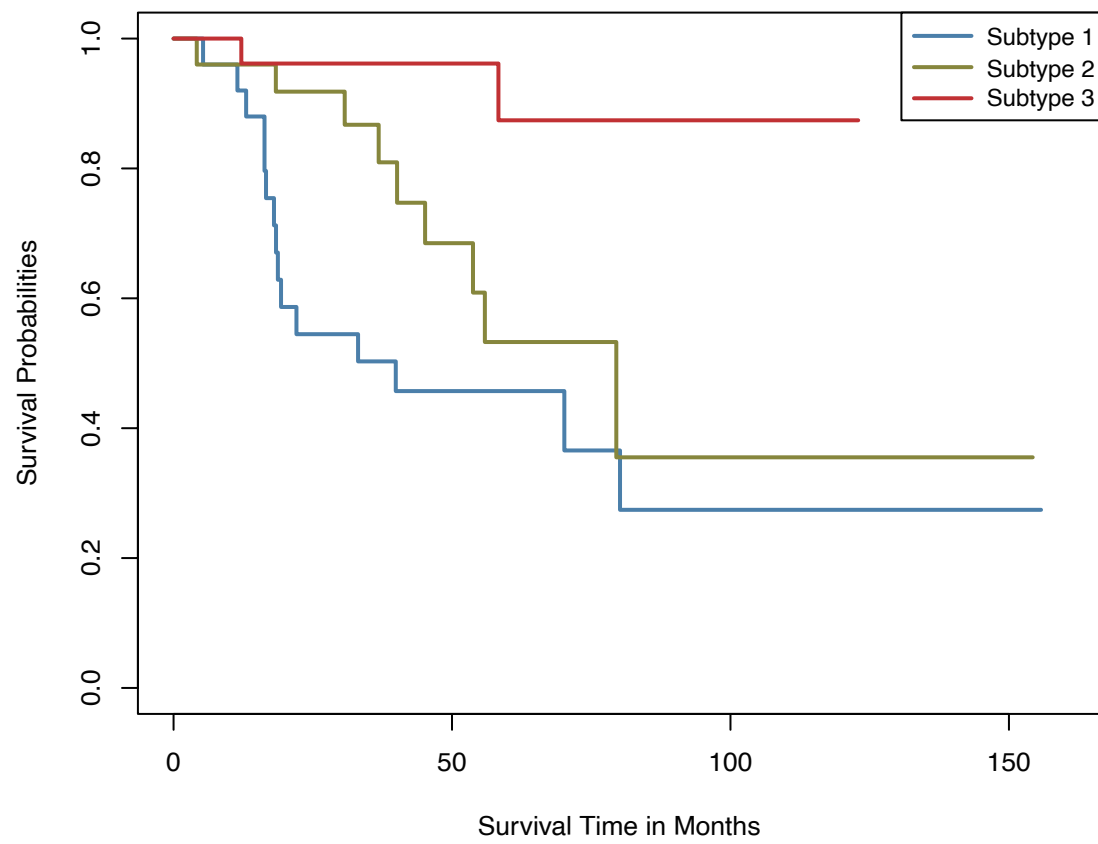

Supplement: Supplementary file 10 — Additional file 10: Fig. S5. Survival analysis of the association between the expression levels of G0S2 and overall survival time in ACC. Kaplan–Meier survival curves for three ACC subtypes which is identified based on the expression levels of G0S2. Patients were classified in three different categories according to mRNA expression of gene G0S2. [file 12859_2021_4029_MOESM10_ESM.pdf]

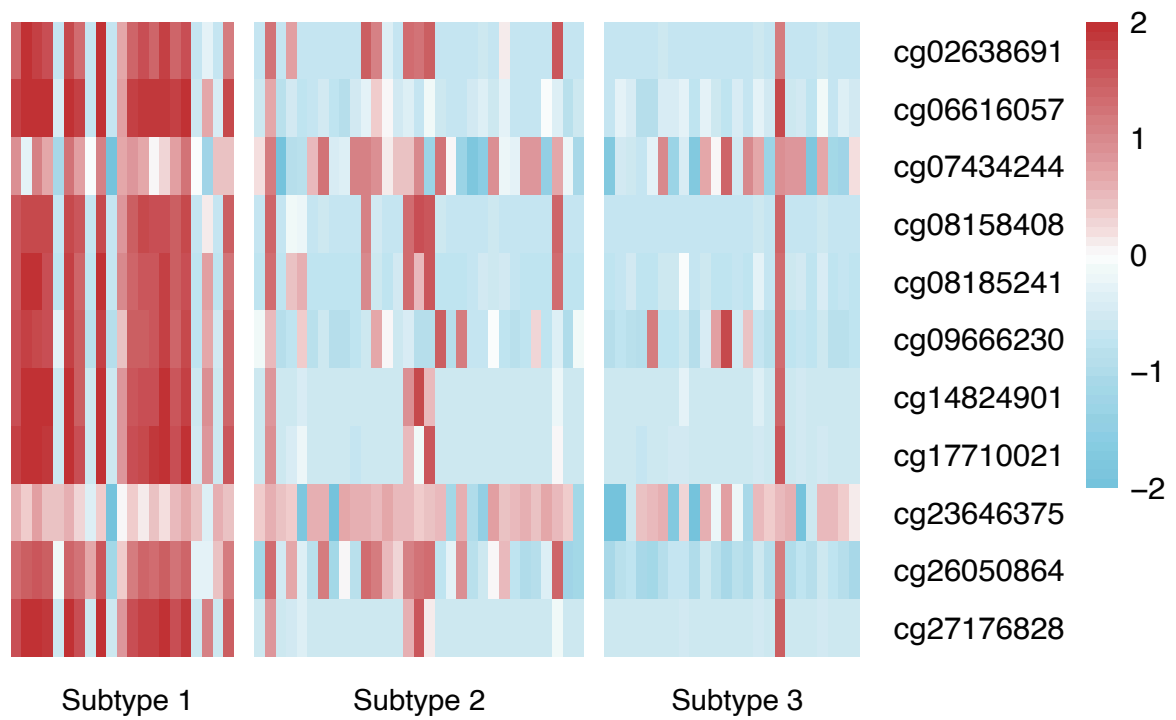

Supplement: Supplementary file 11 — Additional file 11: Fig. S6. The methylation levels at the CpG sites of G0S2. Heatmap of CpG sites’ methylation levels of G0S2 among ACC subtypes that are identified by using RWRF. [file 12859_2021_4029_MOESM11_ESM.pdf]

A

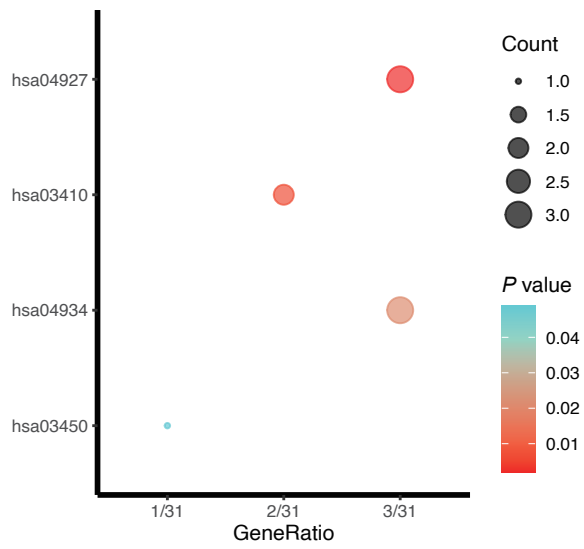

B

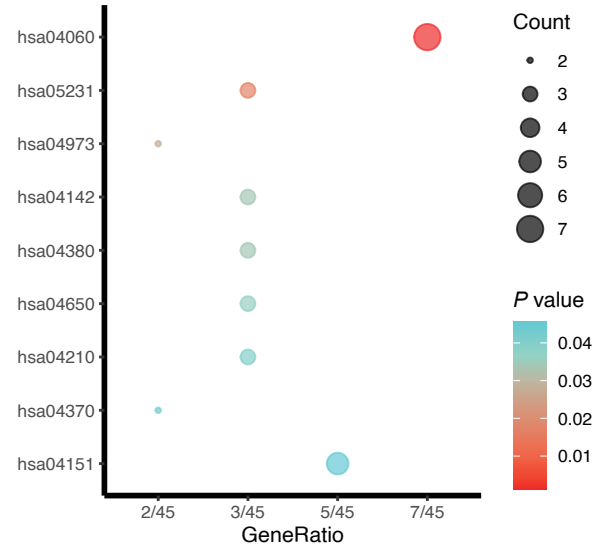

C

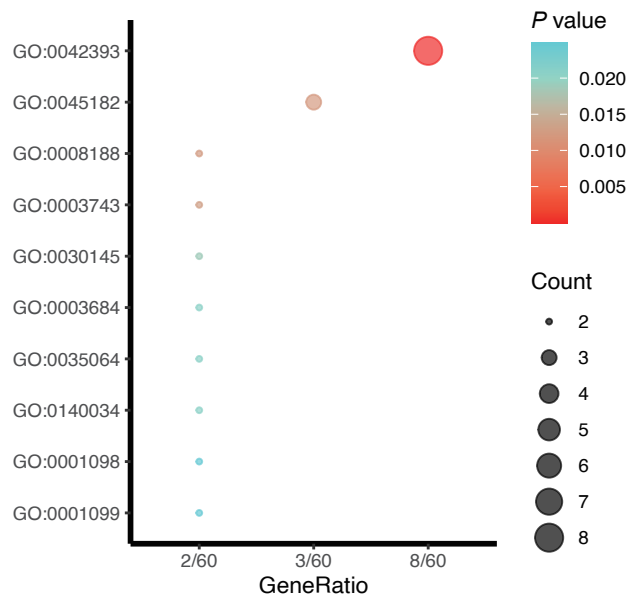

D

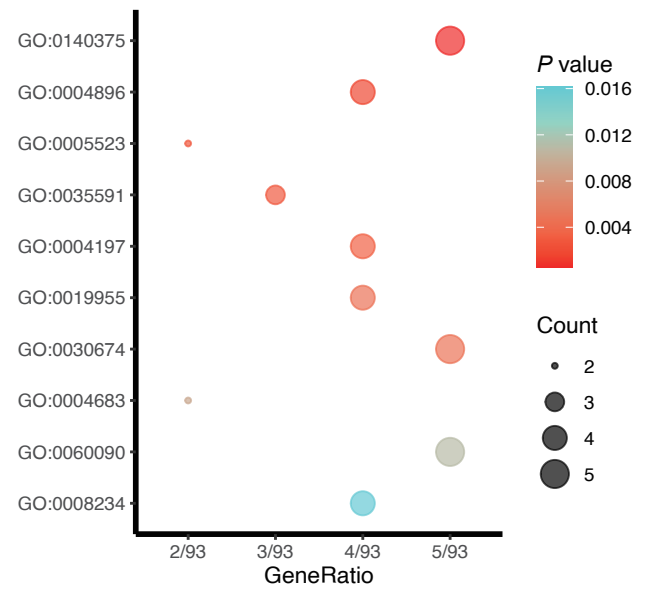

Supplement: Supplementary file 12 — Additional file 12: Fig. S7. KEGG and GOMF enrichment analysis. a, b Dot plots of KEGG enrichment results of subtype1 annotation genes (a) and subtype3 annotation genes (b). c, d Dot plots of GOMF enrichment results of subtype1 annotation genes (c) and subtype3 annotation genes (d). Dot size corresponds to enriched gene quantity. Dot color corresponds to the significance of enrichment. Subtype1 annotation genes and subtype3 annotation genes are these mRNA genes that are significantly differential among ACC subtypes that are identified by using RWRF. [file 12859_2021_4029_MOESM12_ESM.pdf]

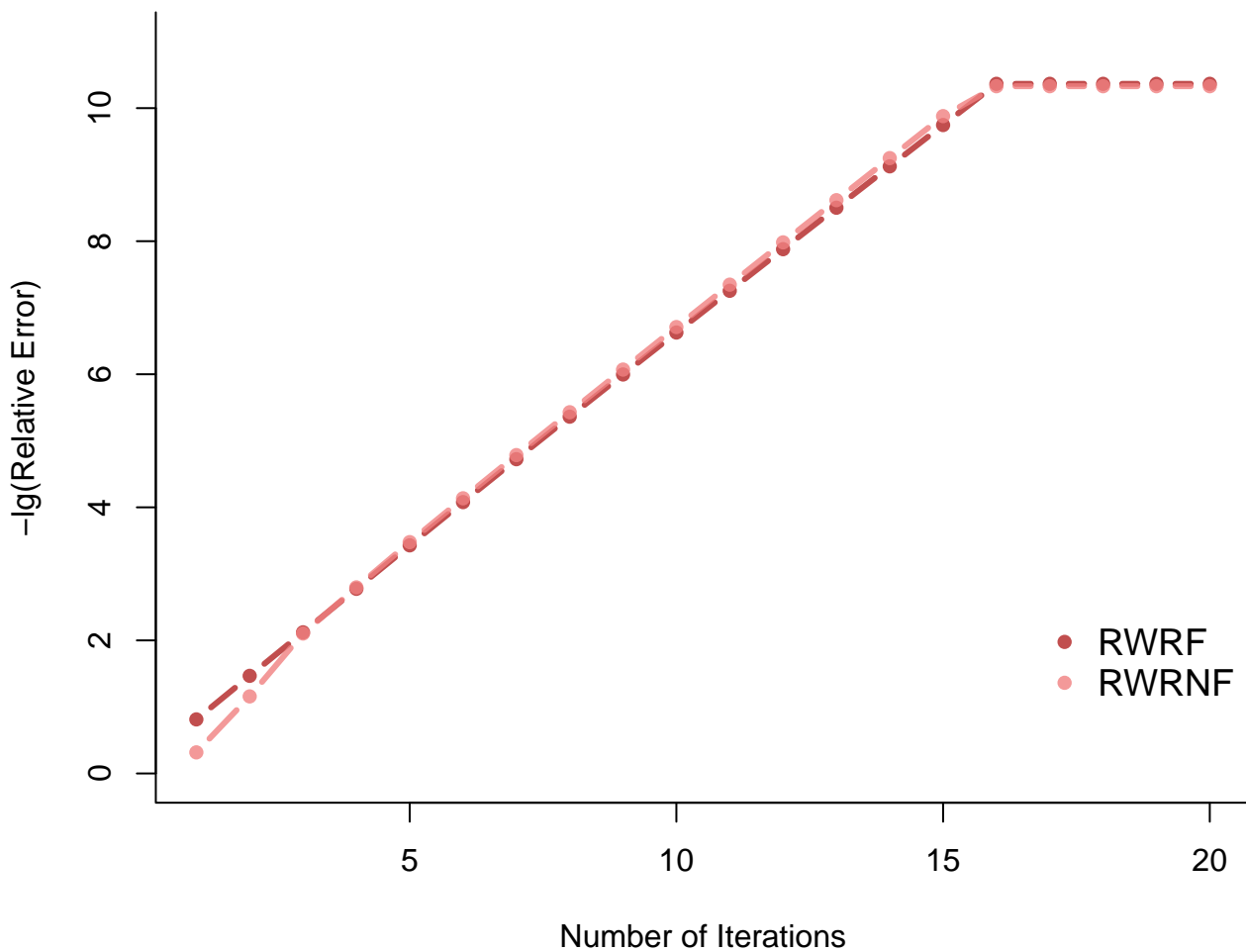

Supplement: Supplementary file 13 — Additional file 13: Fig. S8. Algorithm convergence. The stationary probability distribution $\vec{p}_{stable}$p→stable is usually reached after 16 iterations. [file 12859_2021_4029_MOESM13_ESM.pdf]

A

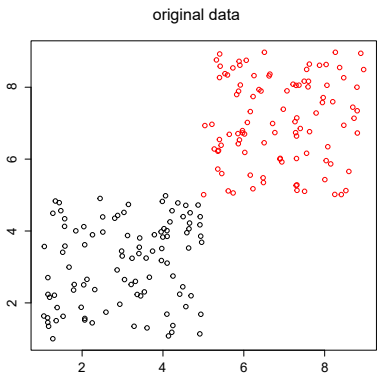

B

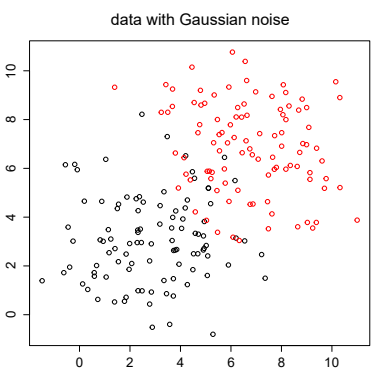

C

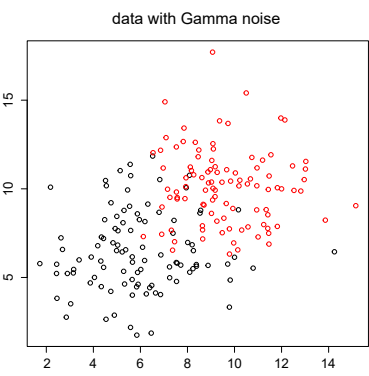

Supplement: Supplementary file 14 — Additional file 14: Fig. S9. Distribution of the simulated data. a The distribution of the original data. b The distribution of the simulated data that are added Gaussian noise with a mean of 0 and a standard deviation of 1.5. c The distribution of the simulated data that are added Gamma noise with shape parameter α = 3 and rate parameter β = 1. [file 12859_2021_4029_MOESM14_ESM.pdf]
